# The Reality of Myoelectric Prostheses: Understanding What Makes These Devices Difficult for Some Users to Control

**DOI:** 10.3389/fnbot.2016.00007

**Published:** 2016-08-22

**Authors:** Alix Chadwell, Laurence Kenney, Sibylle Thies, Adam Galpin, John Head

**Affiliations:** ^1^Centre for Health Sciences Research, University of Salford, Salford, UK

**Keywords:** prosthesis, myoelectric, activity monitoring, control, upper limb, functionality assessment

## Abstract

Users of myoelectric prostheses can often find them difficult to control. This can lead to passive-use of the device or total rejection, which can have detrimental effects on the contralateral limb due to overuse. Current clinically available prostheses are “open loop” systems, and although considerable effort has been focused on developing biofeedback to “close the loop,” there is evidence from laboratory-based studies that other factors, notably improving predictability of response, may be as, if not more, important. Interestingly, despite a large volume of research aimed at improving myoelectric prostheses, it is not currently known which aspect of clinically available systems has the greatest impact on overall functionality and everyday usage. A protocol has, therefore, been designed to assess electromyographic (EMG) skill of the user and predictability of the prosthesis response as significant parts of the control chain, and to relate these to functionality and everyday usage. Here, we present the protocol and results from early pilot work. A set of experiments has been developed. First, to characterize user skill in generating the required level of EMG signal, as well as the speed with which users are able to make the decision to activate the appropriate muscles. Second, to measure unpredictability introduced at the skin–electrode interface, in order to understand the effects of the socket-mounted electrode fit under different loads on the variability of time taken for the prosthetic hand to respond. To evaluate prosthesis user functionality, four different outcome measures are assessed. Using a simple upper limb functional task prosthesis users are assessed for (1) success of task completion, (2) task duration, (3) quality of movement, and (4) gaze behavior. To evaluate everyday usage away from the clinic, the symmetricity of their real-world arm use is assessed using activity monitoring. These methods will later be used to assess a prosthesis user cohort to establish the relative contribution of each control factor to the individual measures of functionality and everyday usage (using multiple regression models). The results will support future researchers, designers, and clinicians in concentrating their efforts on the area that will have the greatest impact on improving prosthesis use.

## 1 Introduction

Statistics relating to the prevalence of limb absence and provision of prostheses are poor. However, data from the United States in 2005 show that ~41,000 people were living there with major upper limb absence (Ziegler-Graham et al., 2008), which equates to 1 in 10,000 people. Furthermore, each year in England ~5–6,000 major limb amputations are carried out (NHS Choices, 2014), of which approximately a fifth are undertaken on the upper limb (Lusardi et al., 2013; NHS Scotland, 2014) and most commonly at the trans-radial (forearm) level (UNIPOD – United National Institute for Prothetics and Orthotics Development, 2010/11). In addition, congenital deformities contribute significantly to the number of people living with upper limb absence, although data on prevalence are somewhat inconsistent (Kyberd et al., 1997; UNIPOD – United National Institute for Prothetics and Orthotics Development, 2010/11; Head, 2014).

Three types of upper limb prostheses are available to people with limb absence: cosmetic prostheses, which are primarily designed to restore appearance and symmetry; and functional prostheses, which are either body-powered via the use of a harness and cables, or electrically powered via rechargeable batteries. Electrically powered prostheses, commonly known as myoelectric prostheses, are controlled by electromyographic (EMG) signals created in the residual musculature, which are picked up by electrodes housed within the prosthetic socket. However, despite the potential offered by myoelectric hands, prosthesis users report these devices to be challenging to control (Biddiss and Chau, 2007a; Peerdeman et al., 2011; Head, 2014; Engdahl et al., 2015) and to still be limited in function (Biddiss and Chau, 2007a; Peerdeman et al., 2011). These user reports are supported by the results of clinical assessment tests in which upper limb prosthesis users generally perform at less than 50% of the level of their anatomically intact counterparts (Mathiowetz et al., 1985; Grice et al., 2003; Farrell et al., 2005; Kyberd et al., 2009; Metzger et al., 2010; Bouwsema et al., 2014; Sobuh et al., 2014). Unsurprisingly, passive-use and rejection of myoelectric prostheses have been reported as problems (Biddiss and Chau, 2007b), leading to over-use injuries of the intact limb (Jones and Davidson, 1999; Gambrell, 2008; Østlie et al., 2011).

In response to user feedback, attempts have been made to improve the control of myoelectric prostheses. Since current clinically available devices are “open loop” with respect to the user, promoting reliance on visual feedback, recent advances have frequently focused on providing users with tactile feedback (Antfolk et al., 2013; Kim et al., 2014; Tee et al., 2015; Oddo et al., 2016; Xu et al., 2016). However, Saunders and Vijayakumar (2011) demonstrated that, although the introduction of feedback can improve control of myoelectric prostheses, other characteristics of the prosthesis may be equally, or even more important in determining the ability of the user to control their prosthesis. In their study, participants demonstrated that when using a “perfect” fast-responding prosthesis they were able to demonstrate good levels of control over grip force even in the absence of any feedback; however, in the presence of “*uncertainty*” as to how the hand would react (presented in the form of random delays in prosthesis response time), their control of the prosthetic hand decreased. Saunders concluded that if the central nervous system (CNS) is able to produce accurate predictions of anticipated prosthesis behavior (forward models), then reliance on feedback from the hand is reduced. Saunders and Vijayakumar (2011) also noted that a degree of “*uncertainty*” was an inherent part of myoelectric prosthesis use. This observation was further investigated by Head (2014) who identified that the standard method for housing electrodes in prosthetic sockets can result in EMG signal artifacts, or loss of electrode contact with the skin, leading to “*unpredictability*” in the response of the prosthesis to muscle contractions. Finally, despite recent findings in anatomically intact subjects (Terlaak et al., 2015) challenging the assumption, there is a widely held belief that there is a relationship between the level of skill in producing the required EMG signals and prosthesis control.

In summary, despite technological advances, control of myoelectric prostheses remains challenging, leading to device rejection and associated over-use injuries of the intact limb. Introducing feedback into the system is one possible solution to enhance prosthesis control for improved “*functionality*” and “*everyday usage*,” however, research into the different aspects of the prosthesis control chain (e.g., “*unpredictability*” in the system and “*EMG skill*” of the user) may be equally important. Here, we introduce a novel protocol, including purpose-built, portable instrumentation that has been designed for the assessment of these individual factors contributing to feed-forward prosthesis control in relation to aspects of overall upper limb performance. Specifically, the protocol assesses how well a myoelectric user can control their EMG signals (“*EMG skill*”) and how reliably the electrodes pick up the signals (“*unpredictability*”). These outcomes are then related to measures reflecting how close the kinematic and gaze patterns of the user are to healthy norms (“*functionality*”) during performance of a structured multistage manual task, and how often the myoelectric prosthesis is used in everyday life (“*everyday usage*”). It is important to note the separation of these two performance measures. Literature has shown that an increase in upper limb “*functionality*” as assessed using clinical tests may not necessarily correspond to an equivalent improvement in everyday arm use (Bailey et al., 2015). By comparing the control factors against “*functionality*” and “*usage*,” it should be possible to identify which factor(s) has/have the greatest impact on overall performance. Longer-term, researchers, designers, and clinicians can then ensure that their efforts are concentrated on the area(s) that will be of greatest benefit to prosthesis users.

In this paper, we introduce the experimental procedures to characterize key factors contributing to the feed-forward prosthesis control chain, namely skill in controlling the EMG signals (“*EMG skill*”) and “*unpredictability*” in transduction of the EMG signal (between the skin and the electrode). We also describe the measures designed to capture the user’s overall upper limb performance (“*functionality*” and “*usage*”). Initial results of our pilot work and their discussion are included to demonstrate the feasibility of the protocol. Furthermore, we propose a data analysis method to be used in the main study, which attributes variance in measures of performance to one or more elements of the control chain.

As the protocol is complex and involves the description of several experimental setups, we have kept the detail in the main body of the paper to a minimum and make use, where appropriate, of Supplementary Material.

## 2 Methods and Analysis

### 2.1 EMG Skill

The muscle groups used to control the opening and closing of myoelectric hands and their associated neural pathways differ from those used in the anatomical hand (Bongers et al., 2012). It is, therefore, reasonable to assume that opening/closing the hand with this “new” set of muscles in response to a relevant prompt may be less intuitive and require an increase in mental processing time as reflected in an increased response time (Kuiken et al., 2004, 2007). It is also reasonable to assume that practice using this “new” set of muscles to open/close the hand may decrease the response time (Ando et al., 2002; Radhakrishnan et al., 2008).

**Figure 1:**
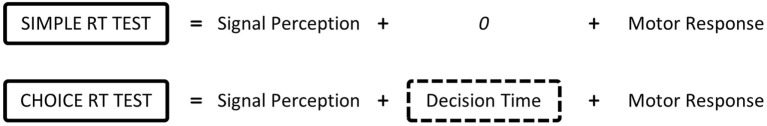
**Donders proposes that reaction times are made up of a series of cognitive and motor processes**. According to Donders’ subtraction method, the choice reaction time minus simple reaction time provides the time taken to decide which muscle to activate based on the stimulus.

To establish the mental processing time to activate the “new” set of muscles, the subtractive method developed in the 1860s by Donders (1869) can be used. Donders proposed the use of different types of reaction time tests to establish the time spent undertaking cognitive and motor processes. Such reaction time tests include the simple reaction time (SRT) test in which participants are aware of the required response before stimulus presentation, and the choice reaction time (CRT) test where the stimulus dictates the required response. Donders segmented the response time into the time taken to perceive the stimulus (signal perception), time taken to decide how to respond (decision time), and time taken to activate the neurons (motor response) (Figure 1). For the SRT test, there is no decision time as the required response is already known and the person is primed to react. Donders also declared that the time for signal perception and motor response does not vary between the tasks. Consequently, he suggested that subtracting the SRT from the CRT provides information as to the decision time to undertake the CRT test, or in this case, how long it takes the person to decide which muscles to activate to operate the prosthesis (“*Decision Time*”). Accordingly, this study uses reaction times measured under two different conditions to characterize the “*Decision Time*,” and associated tests are termed “*Reaction Time Tests*” (see section 2.1.2).

Furthermore, the ability to control the amplitude of the EMG signal using the musculature of the residual limb can be measured through the performance of a series of continuous signal tracking tasks. There are two main types of tracking tasks: static and dynamic. For a static tracking task the subject is required to match their EMG signal to a target amplitude (Alcaide-Aguirre et al., 2013), while a dynamic tracking task involves modulating the amplitude of the EMG signal to match a moving target (Guo et al., 2009; Corbett et al., 2011; Alcaide-Aguirre et al., 2013; Lobo-Prat et al., 2014). Most clinically prescribed myoelectric prostheses are equipped with proportional control, meaning that it is not only important that a user is able to generate a signal strong enough to activate the hand but that they can also modulate the level of the signal to allow for control of the hand speed and the grip force. Dynamic tracking tasks take different forms: some contain a repetitive signal modulation, such as a sinusoidal wave of a set amplitude (Guo et al., 2009), while others vary the amplitude at random (Corbett et al., 2011; Lobo-Prat et al., 2014). For this study, we use a commercially available software package originally designed for the clinical training of myoelectric prosthesis users, which provides us with a means to test user performance in tracking both static and random amplitude modulated targets, using their EMG signal. The approach also allows us to use clinical EMG electrodes (rather than laboratory-standard EMG gel electrodes), thereby reflecting the transduction, signal processing, and amplification used in practice. We term the set of static and dynamic tracking tasks to be “*Tracking Tasks*” (Section 2.1.3).

Details of the number of repeats for each test are provided in Table 1A.

**Table 1A:** **Protocol summary – tests for the assessment of “*EMG skill*”**.

Description	Test	Number of trials
Tests for the assessment of “*EMG skill*.” All undertaken with an “*ideal*” electrode interface condition	Simple reaction time (SRT) – hand opening	2 × practice, 10 × assessed
	Simple reaction time (SRT) – hand closing	2 × practice, 10 × assessed
	Choice reaction time (CRT)	4 × practice (2 × open, 2 × close – random order)
		20 × assessed (10 × open, 10 × close – random order)
	Static tracking task – open signal	3 × assessed
	Static tracking task – close signal	3 × assessed
	Dynamic tracking task – open signal	2 × assessed
	Dynamic tracking task – close signal	2 × assessed
	Dynamic tracking task – both signals	2 × assessed

#### 2.1.1 Electrode Placement

The “*EMG skill*” analysis tests require an “*ideal*” electrode placement on the residual limb to ensure that the participant is able to perform to their best ability. This “*ideal*” placement requires the electrode to be placed in the optimal location, with the optimal gain and good contact with the skin.

Slight variations exist in the methods used to find the optimal location for the electrodes; for this protocol, we use the methods taught to student prosthetists at the University of Salford. Rather than use the participant’s own electrodes, which would necessitate dismantling the prosthetic socket, we use one of two standard electrodes, selected to best match the participant’s own type of electrode (Ottobock 13E200 = 50 or RSL Steeper SEA200). Optimal settings for the selected electrode are found using the clinical assessment tool Myoboy^®^ (Ottobock Gmbh). Initially, the gain for each electrode is set at a mid-level of 3–4. Participants are then asked to repeatedly and consistently contract the muscle to a comfortable level. The electrode is initially placed in the center of the muscle bulk and the signal level is noted. The electrode is then moved in each of four directions (up, down, left, and right) from the starting location, by half an electrodes width. If the amplitude of the signal is greater in any of these new locations, the process is repeated using the new location as the starting point. This is continued until the position with maximum signal amplitude is found, and the location marked using an indelible pencil.

The gain settings are adjusted until the participant is able to comfortably achieve a post-processed signal amplitude, recorded by Myoboy^®^, between 30 and 60 and separation between the two signals greater than 5.^1^ To achieve consistent good contact of the electrodes with the skin, they are bandaged in place using elasticated bandages. The difference between the optimal location and gains, and the location and gains for the participant’s own prosthesis, is noted.

#### 2.1.2 Reaction Time Tests

For these tests, the “*ideal*” electrode placement (Section 2.1.1) is used to control a MyoHand VariPlus Speed (Ottobock Gmbh).

**Figure 2:**
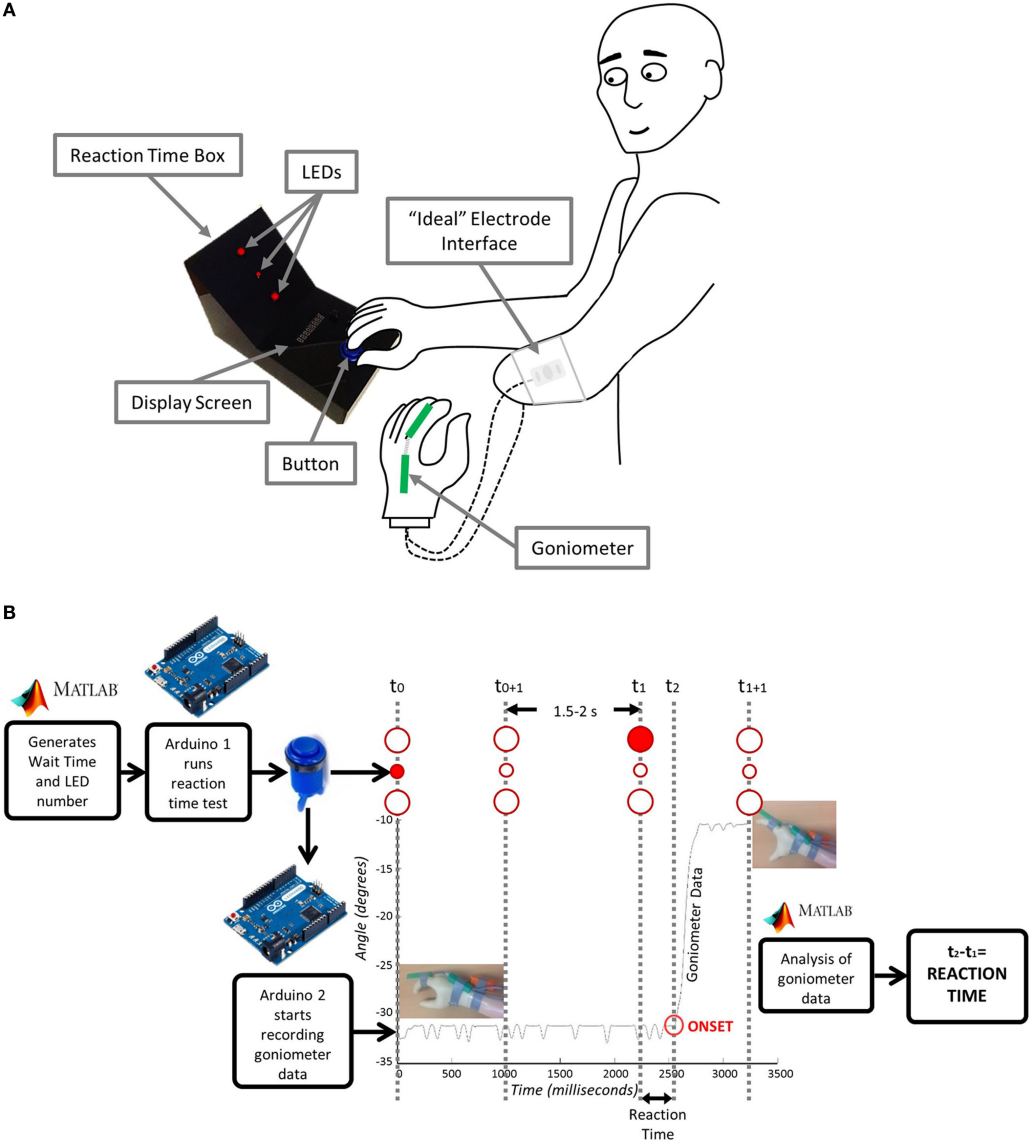
**Reaction time test: (A) Experimental setup and (B) underlying instrumentation**. Matlab generates the wait time and LED number and sends them to Arduino1 which starts the test. The user acknowledges that they are ready by pressing the button. The goniometer begins recording and the central LED lights up for 1 s. After a period of 2.5–3 s, one of the larger LED’s lights up and the user moves their hand. Arduino2 connected to the goniometer sends the movement data to Matlab where it is analyzed and a reaction time is sent back to the user.

A schematic of the experimental setup is shown in Figure 2A. The participant begins each trial with the prosthetic hand in a neutral position. In front of the participant is a custom-made reaction time box with two 10-mm red LEDs serving as stimuli for hand opening (top) and closing (bottom), and one 5-mm red LED in their middle to focus the subject’s attention at the start of each trial. The anatomical hand is placed on a large blue button situated on the bottom portion of the box. The trial begins when the participant indicates that they are ready by pressing the blue button. Each trial then starts with the 5-mm LED illuminating for 1 s to attract the participant’s attention. At a randomly generated time between 2.5 and 3 s (Poliakoff et al., 2013) after the subject pushes the button, one of the 10-mm LEDs will then illuminate for 1 s. Once the 10-mm LED turns on, the participant should then either open (if top LED) or close (if bottom LED) their prosthetic hand in response. For the SRT Test, the subject is aware which LED will illuminate, i.e., which response is required. For the CRT Test, the subject needs to respond with either hand opening or closing, dependent on whether the top or bottom LED is illuminated. For all reaction time tests, an electronic goniometer (Biometrics Ltd) is attached across the proximal knuckle of the index finger to measure the movement of the prosthetic hand, thereby allowing for identification of the onset of hand opening or closing.

Details of the instrumentation used in SRT and CRT trials are shown in Figure 2B. The reaction time box and goniometer are controlled via Arduino Leonardo boards (www.arduino.cc) communicating over serial with Matlab (The Mathworks Inc.). The wait time and LED number are sent from Matlab to Arduino1 to start the test. Arduino1 waits for acknowledgment that the user is ready, based on their button press. Arduino1 then initiates recording of the goniometer data on Arduino2 and controls the LEDs on the reaction time box. Matlab then analyzes the goniometer data establishing the reaction time, which is sent back to Arduino1 and displayed to the participant. A T9545 goniometer adaptor (Thought Technology Ltd.) and TT Sensor Isolator ST9405AM (Thought Technology Ltd.) are used to interface between the goniometer and Arduino2.

#### 2.1.3 Tracking Tasks

This test uses commercially available assessment tools from Ottobock Gmbh that are routinely used in clinical care. The Myoboy^®^ hardware is designed to measure the signal from the clinical electrodes and send it to a computer. Using the PAULA (Prosthetist’s Assistant for Upper Limb Architecture) software, the signal can be viewed and the user can then undertake activities to train and improve signal control. The “*ideal*” electrode placement (Section 2.1.1) is used with the electrodes connected to the Myoboy^®^ hardware. Two different aspects of the PAULA software are used, one for the “*static tracking task*” and one for the “*dynamic tracking task*.”

**Figure 3:**
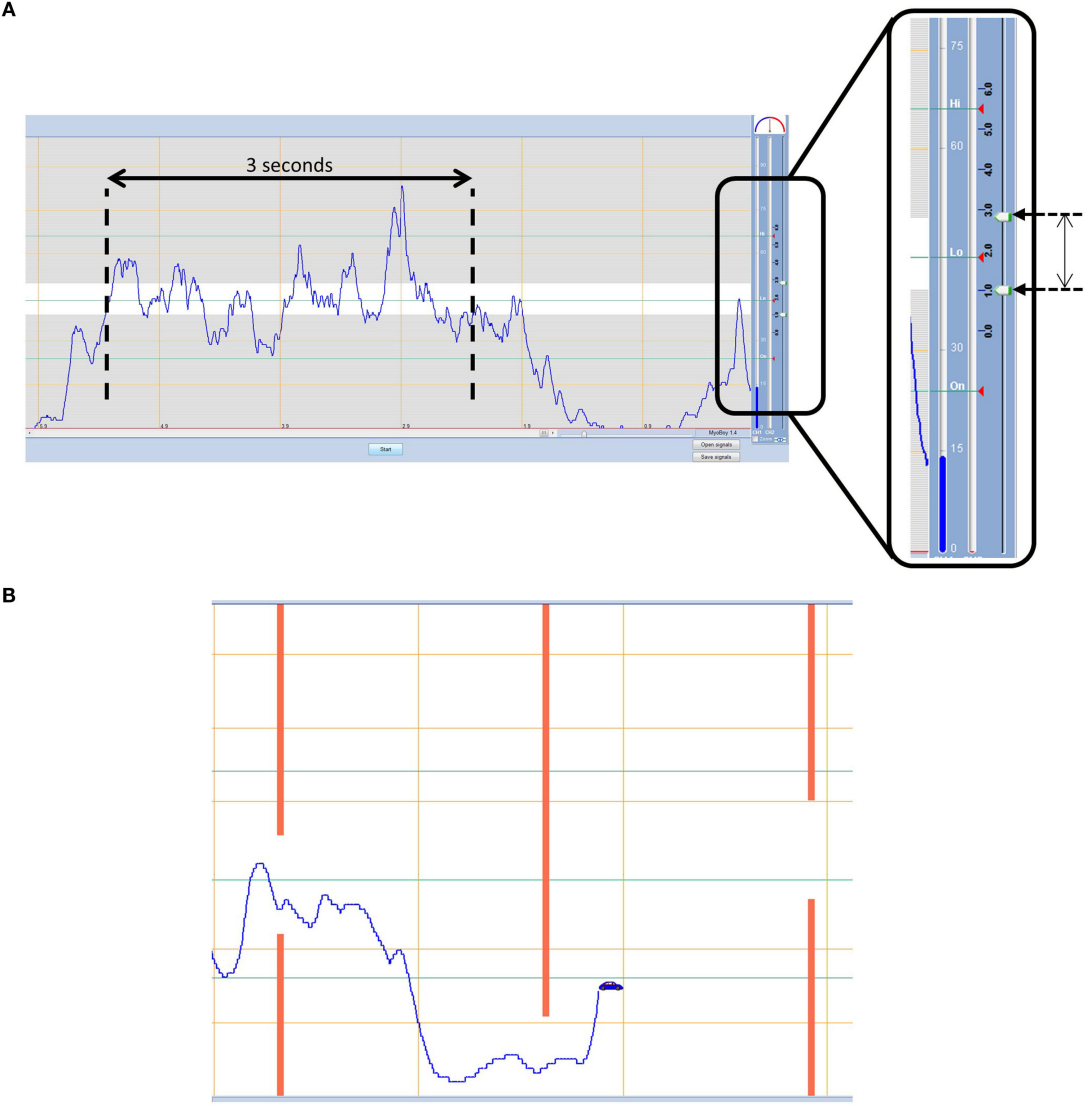
**(A) Static tracking task** – participants must aim to keep their signal within the boundaries for a 3 s period. **(B) Dynamic tracking task** – participants must navigate a car through gaps in approaching walls using muscle contraction and relaxation.

The “*static tracking task*” uses the myo-testing signal visualization screen (Figure 3A). The boundary lines within this screen are adjustable and in this protocol are set to 39 and 51; these values were determined through pilot work as a level that is sufficiently challenging for the more skilled participant, yet somewhat achievable for the least able. The participant is given three contraction attempts to keep their signal amplitude within the boundaries for each muscle. Each contraction is 3 s long from the moment the signal first crosses the lower boundary line. Participants are scored on the percentage of time the signal remains within the boundaries.

The “*dynamic tracking task*,” on the other hand, uses the training “car game” within PAULA. The task involves steering a car through gaps in approaching walls that fluctuate in height (Figure 3B). The game level is set in the middle of the available options at 5, and the training time is 1 min which during pilot work proved to be long enough that no one achieved a perfect score, without being too long that people who were struggling stopped trying. The height of the car is controlled using the EMG signal; muscle contraction elevates the car on the screen and muscle relaxation drops the car to the bottom of the screen. Beginning with the hand-open signal the participant must steer the car through the approaching gaps that cycle between being high and low (contraction and relaxation). Participants are given two attempts to get the best score they can achieve, defined as the percentage of gaps successfully passed through without crashing (Part 1a). The task is then repeated for the hand-close signal (Part 1b). Finally, the participant must control two cars at once using both signals (Part 2). During part 2, the cars are set up so that when one muscle is contracted the other one should be relaxed, assessing the ability of the participant to separate their signals, while cycling between hand opening and closing.

### 2.2 Effects of Electrode Interface Condition on EMG Signal Transduction

Good electrode contact with the skin is required for reliable transduction of the EMG signal. Prosthesis electrodes (known as myoelectrodes) are “dry” metal electrodes housed in a plastic case; a small gap in the prosthesis socket is designed to house the myoelectrode; two rubber projections extend from each end of the casing, which locate within pre-manufactured slots in the socket walls. Although a surprisingly neglected area, it is established that the design of prosthetic sockets and associated electrode housings can lead to problems in the transduction of the EMG signal. For example, applied load may cause the socket to move relative to the residual limb and, hence, produce signal artifacts, or electrode contact may be lost altogether (Head, 2014). Furthermore, it is possible that re-donning of the socket may lead to the electrodes moving from the optimal location (see Electrode Placement), leading to crosstalk from other muscles. These factors constitute “*unpredictability*” in the transduction of the EMG signal, leading to “*uncertainty*” as to the response of the prosthetic hand to neural commands.

**Figure 4:**
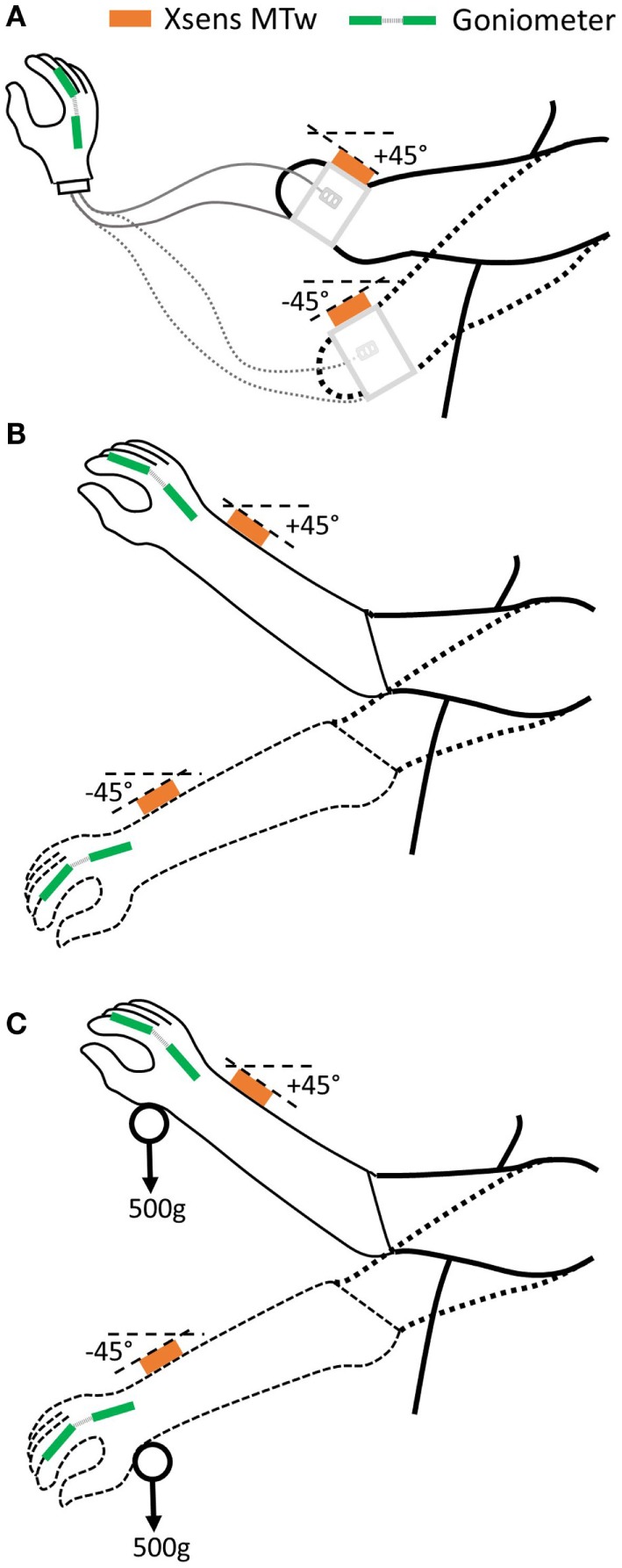
**Three electrode interface conditions will be assessed. (A)** “Ideal”: no socket, electrodes bandaged to residual limb, **(B)** “Normal”: prosthetic socket-housed electrodes, and **(C)** “Additional load”: prosthetic socket + 500 g load.

Our protocol builds on previous work in this area (Head, 2014) to assess two key aspects of “*uncertainty*”: (1) whether the hand responds when the user desires it and (2) whether the hand activates unexpectedly. Specifically, to assess the impact of the socket-housed electrode fit on these two “*uncertainty*” measures, participants complete a set of tasks with the forearm held at two different angles, under three electrode interface conditions (Figure 4): (1) “*Ideal*” – the electrodes are placed in the optimal position on the residual limb and held in place using elastic bandage as in Section “2.1.1” (Figure 4A). The electrodes are connected to the MyoHand VariPlus Speed (Ottobock Gmbh) as in Section “2.1.2,” which is sat on the table top; using this method, there should be minimal or no movement of the electrodes in relation to the skin. (2) “*Normal*” – the prosthesis is worn as normal, and the electrodes are housed in the prosthetic socket (Figure 4B). From this part of the study onward, the participant uses their own prosthesis with the electrode location and gain settings which they would use in everyday life. (3) “*Additional load*” – the prosthesis is worn as normal; however, an additional 500 g load is strapped to the hand to simulate the weight of an object, such as a full jar (Figure 4C).

Tasks are undertaken with the arm in postures that are representative of those encountered during daily activities, such as reaching to a shelf, or down into a drawer, corresponding to ~45° above and below the horizontal. Forearm angles from the horizontal are measured using an inertial measurement unit (IMU). For this study, an Xsens MTw sensor (Xsens Technologies B.V.) is used. The IMU is placed on the back of the wrist just proximal to the ulnar styloid. The *x*-axis is aligned along the forearm axis pointing toward the hand. For our pilot work, a proprietary algorithm was used, which outputs orientation components based on Euler angles (XYZ earth fixed); however, in the longer-term, this will be replaced with an algorithm that calculates the orientation of the *x*-axis relative to gravity (Sun et al., in press).

The set of tasks performed at each of the two arm orientations, for each of the three electrode interface conditions are described in the following two sections.

#### 2.2.1 Reaction Time Tests

The impact of the electrode interface conditions on variability in reaction times is assessed using the equipment described in 2.1.2 above. Participants begin with the “*ideal*” electrode interface; the simple reaction time (SRT) test is undertaken at each of the two arm postures. The test is then repeated for the other two interface conditions (“*Normal*,” “*Additional Load*”) at each of the two arm postures. The number of repeats is detailed in Table 1B. The spread in reaction times is compared across the electrode interface conditions, between the “*ideal*” interface and the two socket-housed electrode conditions (“*Normal*,” “*Additional Load*”).

**Table 1B:** **Protocol summary – tests for the assessment of “*Unpredictability*”**.

Description	Test	Arm position	Number of trials
Tests for the assessment of “*unpredictability*” introduced by the electrode interface condition. All tests are repeated for each interface condition (“*Ideal*,” “*Normal*,” and “*Additional Load*”)	Simple reaction time (SRT) – open signal	45° above horizontal	10 × assessed using “*ideal*” interface, 5 × assessed using “*normal*” and “*additional load*”
	Simple reaction time (SRT) – close signal	45° above horizontal	10 × assessed using “*ideal*” interface, 5 × assessed using “*normal*” and “*additional load*”
	Simple reaction time (SRT) – open signal	45° below horizontal	10 × assessed using “*ideal*” interface, 5 × assessed using “*normal*” and “*additional load*”
	Simple reaction time (SRT) – close signal	45° below horizontal	10 × assessed using “*ideal*” interface, 5 × assessed using “*normal*” and “*additional load*”
	Transition – hand open	from 45° above horizontal to 45° below	6 × assessed using “*ideal*” interface, 3 × assessed using “*normal*” and “*additional load*”
	Transition – hand closed	from 45° above horizontal to 45° below	6 × assessed using “*ideal*” interface, 3 × assessed using “*normal*” and “*additional load*”
	Transition – hand open	from 45° below horizontal to 45° above	6 × assessed using “*ideal*” interface, 3 × assessed using “*normal*” and “*additional load*”
	Transition – hand closed	from 45° below horizontal to 45° above	6 × assessed using “*ideal*” interface, 3 × assessed using “*normal*” and “*additional load*”

#### 2.2.2 Transitioning between Arm Postures

Transitions from one posture to another may, in the case of a poor fitting socket, cause an EMG artifact and, hence, cause the prosthetic hand to open or close when the user does not desire it (Head, 2014). Such an event could lead to the user dropping or squashing an object. Therefore, between each set of “*reaction time tests*” (see Section 2.2.1), prosthetic hand posture is recorded as the arm moves between the two arm postures. The hand begins each transition either completely open or completely closed, and prosthetic hand posture is recorded throughout the transition using the goniometer (see Section 2.1.2); any undesired activation, i.e., opening or closing of the hand is recorded.

### 2.3 Functionality Assessment

Upper limb prosthesis user functionality is typically appraised using an appropriate, validated assessment tool, such as the Southampton Hand Assessment Procedure (SHAP) (Light et al., 2002). In common with a number of other clinical tests, functionality is evaluated on the time taken to successfully complete specific tasks. Faster completion times are assumed to correspond to higher levels of functionality. Although the duration of task performance is one measure of functionality, it provides no information on how tasks are completed. A number of studies have shown that combining several outcome measures provides a more complete picture of the functional abilities of prosthesis users (Hill et al., 2009; Wright, 2009; Lindner et al., 2010; Bouwsema et al., 2012). Kinematic outcome measures and gaze behavior can be recorded during the performance of multistage tasks to provide information that complements speed of performance measures. It has previously been shown that performance characterized using these measures clearly differentiates amputees from anatomically intact controls (Bouwsema et al., 2012; Sobuh et al., 2014).

When faced with a novel task, young children are known to try a number of different movement trajectories, allowing the CNS to build a representation of the optimum trajectory (Schneiberg et al., 2002). When faced with structured multistage manual upper limb tasks, novice prosthesis users have been shown to demonstrate similar trends (Major et al., 2014). During the first few task attempts, variability in the linear acceleration patterns of the forearm is high; however, after practice with the prosthesis, variability has been shown to decrease (Sobuh, 2012). Moreover, Bouwsema et al. (2010) demonstrated that prosthesis users demonstrate a later onset of hand opening during reach-to-grasp movements than anatomically intact subjects, and a plateau in the hand aperture between opening and closing around the object.

Furthermore, previous studies undertaken by Bouwsema et al. (2012) and Sobuh et al. (2014) have shown that the gaze behavior of inexperienced prosthesis users differs from that of anatomically intact controls, however, with practice gaze patterns approach those of controls. A more functional user would be expected to demonstrate a larger number of “look-ahead-fixations” and spend less time concentrating on the prosthetic hand. In a multistage task, “look-ahead-fixations” involve gaze fixation on an area of the task critical to a future task component (such as looking at the object to be grasped, or the location it will be moved to while completing the reach, rather than concentrating on the hand). Fewer transitions between areas of interest (AOIs, e.g., hand, grasp area of the target) would also be expected. Interestingly, participants who self-report rarely using their devices in everyday life have been shown to demonstrate more gaze transitions, irrespective of their functional ability with the device (Bouwsema et al., 2012). Prosthesis users are reliant on visual feedback, as such it would be expected that patterns in gaze behavior may be related to a person’s knowledge as to how their hand will respond. If a participant cannot accurately predict the response of their prosthesis, it is possible that this will be reflected in the number of gaze transitions.

We, therefore, assess participants’ performance with their prosthesis using a structured multistage manual task, which involves the reaching for, grasping, then placing and releasing of a cylinder in a tube. Three levels of task difficulty are available to the participants (as described below). Performance is then characterized based on number of successfully completed trials and task difficulty, time to complete the task, aperture onset delay, plateau time during reach-to-grasp, kinematic variability in movements (using accelerometry) and gaze behavior over successive trials.

#### 2.3.1 Task Design

Previous work has suggested that certain movements are prone to cause users with poor fitting sockets particular difficulties in prosthesis control, possibly as a result of artifacts caused by electrode movement in relation to the skin or separation from the skin (Head, 2014). These include movements that would be achieved through “pronation” or “supination” in anatomically intact participants. A set of three multistage unilateral tasks (“*cylinder tasks*”) have been developed (termed “*tasks A–C*”), the harder of which (“*tasks B and C*”) encompass these movements and, hence, present a significant challenge to some participants. Each participant attempts 10 trials (Table 1C) of 2 of the 3 tasks, as follows. All participants begin with the medium difficulty level (“*task B*”). Using the prosthesis, participants reach to grasp a cylinder (dia. 52 mm, length 200 mm, weight ~350 g), lift and rotate it through 90° to the horizontal, place it into a horizontally orientated tube (inner dia. 64 mm, length 100 mm), and then release it returning their hand to the starting position. Participants who have a prosthesis with a wrist rotator are asked not to use this function during completion of any of the manual tasks. If the participant is successful in completing over 80% of the trials without dropping the cylinder, they move to “*task C*” in which the tolerance between the cylinder (dia. 52 mm, length 200 mm, weight ~350 g) and the tube (inner dia. 58 mm, length 100 mm) is reduced. If they are unsuccessful in completing 80% of the medium difficulty trials (“*task B*”) they perform the easier task (“*task A*”), in which the cylinder is placed vertically into a vertically orientated target tube with the same dimensions as “*task B*” (inner dia. 64 mm, length 100 mm).

**Table 1C:** **Protocol summary – tests for the assessment of “*Functionality*” and “*Everyday Usage*”**.

Description	Test	Number of trials
Tests for the assessment of “*functionality*” and “*everyday usage*”. All undertaken with a “*normal*” electrode interface condition	Cylinder task – Task B	10 × assessed
	Cylinder task – Task A or Task C	10 × assessed
	Activity monitoring	1 week (7 days)

As before, participants wear sensors allowing kinematics to be assessed and an eye tracker to record gaze behavior. IMUs (Xsens MTw) are worn on the wrist of the prosthesis and on the chest,^2^ an electronic Goniometer (Biometrics Ltd.) is worn across the proximal knuckle of the index finger, and participants wear a Dikablis Professional Eye Tracker system (Ergoneers). All data are sampled at 50 Hz. The three systems are synchronized using an arcade style button.

#### 2.3.2 Performance Evaluation

To score performance, the task is split into five movement components and the participants gain points for each section they complete successfully. Points are gained for successful completion first time of (1) reach-to-grasp, (2) lifting and rotating the cylinder, (3) placing the cylinder all the way into the tube, (4) releasing the cylinder, and (5) returning to the start position. Half points are allocated if the movement component was completed after a second attempt (provided the cylinder was not dropped or knocked over). Additional points are added to the total to reflect the level of difficulty the subject was able to perform the task at (0, easy task “*A*”; 1, medium task “*B*”; 2, difficult task “*C*”). The participant starts and ends the movement with their hand on an arcade style button from which timestamps are generated, allowing for task duration to be calculated.

#### 2.3.3 Quality of Movement

Quality of movement encompasses both the pattern of hand aperture during reach and the movement variability throughout the task. It is possible to determine the end of the reach phase by analyzing the goniometer data. When the task begins, the hand is completely closed; the hand then opens, before closing again around the cylinder to generate a “*transport plateau*” as the object is transported. It has been shown that prosthesis users demonstrate a delay in opening the hand at the start of reach, demonstrated by an “*onset plateau*,” and a delay between opening and closing the hand, termed the “*reach plateau*” (Bouwsema et al., 2010). The start of the “*transport plateau*” is taken as the end of the “reach-to-grasp” component of the task. By segmenting the “reach-to-grasp” component of the task, the delay in onset of hand movement (the length of the “*onset plateau*”) is calculated as a percentage of the “reach-to-grasp,” and the length of the “*reach plateau*” is calculated as a percentage of the “reach-to-grasp.” Furthermore, using the wrist-mounted IMU, the kinematic variability in the linear acceleration of the forearm between tasks is assessed using the methods developed by Thies et al. (2009).

#### 2.3.4 Gaze Behavior

For the purposes of analyzing the eye tracking videos, the task area is split into six AOIs: (1) start point (button), (2) prosthetic hand, (3) “grasp critical” area (GCA) (bottom half of the cylinder for “*tasks A and B*,” top half for “*task C*”), (4) other “location critical” half of the cylinder (LCA) that is required to be placed into the tube, (5) tube, and (6) LED. The percentage of time spent looking at each AOI is calculated, alongside the number of times that the gaze location transitions between each of these areas. Finally, the percentage of time spent looking at areas of the task relevant to subsequent components of the task (“look-ahead-fixations”) is calculated for each point in the task (e.g., the cylinder and tube during “reach-to-grasp,” or the tube during manipulation and transport).

### 2.4 Everyday Usage

Current methods of quantifying everyday prosthesis use involve self-report (Roeschlein and Domholdt, 1989; Sherman, 1999; Gallagher and MacLachlan, 2000; Raichle et al., 2008), which is known to be prone to recall and bias errors (Metcalf et al., 2007; Brown and Werner, 2008). Accelerometer-based activity monitoring (Noorkõiv et al., 2014) provides an opportunity to observe actual prosthesis use outside of the clinical environment; however, to date no studies have been published on a cohort of upper limb prosthesis users. We have adapted a protocol developed for stroke patients (Bailey et al., 2015). This research involved participants wearing an activity monitor (Actigraph GT3X+) on each of their wrists while they went about their normal daily activities. The Actigraph monitors provide continuous logging of raw accelerometer data (sampled at 30 Hz). The data are downloaded using proprietary software, filtered and down sampled to 1 Hz. The processed data are expressed as activity counts (0.001664 g/count) (Actigraph Corp, 2015), which are converted into vector magnitudes (sum of the counts along each axis $\sqrt{x^{2}+y^{2}+z^{2}}$). For each second of data, Bailey et al. (2015) combined the vector magnitudes from each of the two wrist worn monitors (dominant and non-dominant arm) to inform on the magnitude of activity across both arms, expressed as the “*bilateral magnitude*” (VM_Dom_ + VM_NonDom_), and the contribution of each arm to the activity, expressed as the “*magnitude ratio*” [ln(VM_NonDom_ + VM_Dom_)].

Bailey found that in healthy, anatomically intact controls, the median “*magnitude ratio*” was around zero (symmetrical bilateral arm use); however, in the stroke cohort, the “*magnitude ratio*” was skewed toward unilateral Non-Paretic (unaffected) arm use. In general, participants in the stroke cohort who demonstrated higher levels of functionality [according to the Action Research Arm Test (McDonnell, 2008)] also demonstrated “*magnitude ratios*” closer to those of the healthy control subjects; nevertheless, a third of participants demonstrated a median “*magnitude ratio*” representing unilateral non-paretic arm use, regardless of their functionality with the paretic arm.

For our study, the activity monitors are placed on the anatomical wrist and the wrist of the myoelectric prosthesis. The monitor is not transferred to other prostheses the participant may wear (e.g., body-powered), as only the times when the myoelectric prosthesis is in use are of interest to this study. Participants are invited to wear the monitors for 1 week.

### 2.5 Pilot Study

#### 2.5.1 Recruitment

**Figure 5:**
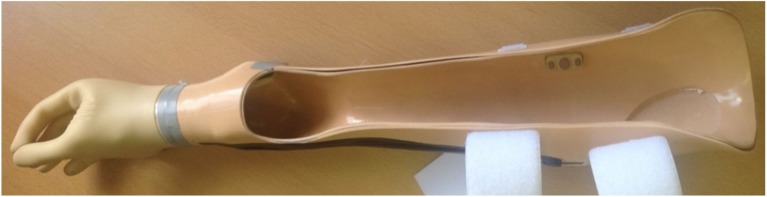
**Prosthesis simulator for use with anatomically intact subjects**. The socket is designed to fit over the forearm and fist. Straps allow the socket to be tightened to the persons arm. It is not possible to tailor electrode placement to each person.

The purpose of this pilot study was to assess the robustness and feasibility of the protocol before undertaking the main study with a cohort of myoelectric prosthesis users. Ethical approval was granted by the University of Salford School of Health Sciences Research Ethics committee (*REF: HSCR 15-130*) to pilot the above protocol with anatomically intact subjects using a prosthesis simulator designed to fit over their intact arm (Figure 5), and myoelectric prosthesis users recruited from the University of Salford Prosthetics and Orthotics Professional Patient Database. Inclusion criteria for the latter were (1) an amputation or congenital limb loss at the trans-radial level, (2) owning a myoelectric prosthesis, and (3) over 18 years of age. Exclusion criteria were (1) bilateral limb loss, (2) injury to the residual limb at the time of testing, and (3) using single site muscle control.

#### 2.5.2 Data Analysis

##### 2.5.2.1 Factors Affecting Prosthesis Control

As described above (see Sections 2.1 and 2.2), “*EMG skill*” and “*uncertainty*” both affect control of the prosthesis. Multiple variables are generated as part of this protocol that characterize these factors, and which must be combined into overall scores for skill in controlling the EMG signals (“*EMG Skill Score*”), and “*uncertainty*” introduced by the electrode interface (“*Uncertainty Score*”). For this reason, the pilot study data were reduced to ordinal data.

Specifically, the “*EMG Skill Score*” is devised of the reaction time difference between the choice and simple reaction times (termed “*Decision Time*,” see Section 2.1), and the scores from the “*Tracking Tasks*.” To ensure that the reaction times reported were not biased by early or late reactions, any responses faster than 100 ms or slower than 1000 ms were excluded from the analysis (Press et al., 2005). When combining the three scores contributing toward “*EMG Skill*,” the “*Dynamic Tracking Task*” score was given a higher weighting, since accurately proportionally controlling a dynamic and noisy signal poses a greater challenge than the “*Reaction Time Tests*” or the “*Static Tracking Task*.”

A combined score for the “*uncertainty*” introduced by the electrode interface must also be generated (“*Uncertainty Score*”). This is an ordinal score based on the reaction time spread across the conditions highlighted in Section 2.2.1 and the number of unwanted activations during the transitions (see Section 2.2.2).

##### 2.5.2.2 Prosthesis Functionality and Everyday Usage

The variables characterizing prosthesis “*functionality*” and “*usage*” were split into four key areas as described in Sections “2.3” and “2.4.” Of the three possible functional tasks (easy “*A*,” medium “*B*,” and hard “*C*”), all participants attempted two that were analyzed independently.

Initially, a basic performance evaluation was undertaken. A “*performance score*” was generated based on the scores for the completed movement components of the task (see section 2.3.2) and the task duration (in seconds). Participants were penalized up to a total of 1 s for movement components they failed to complete successfully.

For all trials where the participant completed the “reach-to-grasp” component of the task, the hand aperture profile was analyzed to establish the percentage of reach consumed by the “*reach plateau*” period and the “*delay plateau*.” Using the methods developed by Thies et al. (2009), variability in the linear acceleration of the forearm was also calculated, for both the “reach-to-grasp” component and the full task.

Analysis of the eye tracking data used a coding scheme to record the AOIs on which the gaze was concentrated for every frame, allowing for the time spent in each AOI and the number of transitions between AOIs to be calculated. Furthermore, the time spent looking ahead to the next component of the task was calculated.

Finally, analysis of the activity counts recorded by the Actigraph activity monitors allowed for the calculation of the “*bilateral magnitude*” and “*magnitude ratio*” using the methods described in section “2.4.” By combining the raw data with the activity diary, it was also possible to establish the wear time of the prosthesis. For the purposes of this study, the results are reported based on the data recorded throughout the week, irrespective of whether the prosthesis was worn. However, to allow fair comparison of the “*magnitude ratio*” between prosthesis users and stroke patients (Bailey et al., 2015), analysis was also undertaken based only on the periods when the prosthesis was worn. For this secondary analysis, overnight removal of the prosthesis was excluded based on visual assessment of the raw accelerometer data and activity counts from the monitor worn on the prosthesis. Data were excluded from the last activity count on 1 day until the first count on the next day (activity count spikes during these non-wear periods, lasting less than 1 min with at least 10 min of non-use either side, were also excluded). If visual analysis of the raw data showed long periods (>1 h) of no prosthesis activity during the day, these periods were also excluded based on the activity counts, with the assumption that the prosthesis was removed. For more information on the visual analysis, please see the Supplementary Material. Similarly to Bailey’s data, the median “*magnitude ratio*” was reported to avoid the effects of skewness.

##### 2.5.2.3 Relationships between Factors Affecting Prosthesis Control and Functionality/Usage

The early pilot work was not intended to draw conclusions on the relationship between the different factors. However, the main study will aim to establish how measures of “*functionality*” and “*everyday usage*,” can be explained by the factors affecting myoelectric prosthesis control. Using multiple regression techniques, factors affecting prosthesis control (i.e., “*EMG skill*” and “*uncertainty*”) will be related to measures of “*functionality*” and “*everyday usage*,” specifically:

- the sum of the points gained for successful completion of the task,
- the mean “*performance score*,”
- the aperture profile and movement variability during the reaching phase,
- the movement variability during the performance of the full task,
- the percentage of time spent looking at each AOI,
- the number of gaze transitions,
- the percentage of time spent in “look-ahead” gaze fixations, and
- the “*magnitude ratio*” between the two hands during everyday activity.

To further characterize upper limb performance, measures of “*everyday usage*” will be correlated against measures of “*functionality*” collected within the clinic. These may include association of the “*magnitude ratio*” and wear time with:

- the percentage of time spent looking at each AOI,
- the number of gaze transitions, and
- the movement variability during the performance of the full task.

Based on the findings of these analyses, it should be possible to establish the relative contribution of the factors affecting prosthesis control to each measure of “*functionality*” and “*everyday usage*.”

## 3 Initial Pilot Study Results and Discussion

In this section, we use early results from initial pilot work with anatomically intact subjects using a prosthesis simulator and prosthesis users to demonstrate the feasibility of this protocol. Data collected from two prosthesis users (both male, age 44–45, with congenital limb absence, and 1.5–35 years using a myoelectric prosthesis) and one anatomically intact subject using a prosthesis simulator (male, age 21, no experience) are presented.

### 3.1 Data Collection

The data collection period lasted between 4 and 5 h, including breaks, which was longer than desired, however, the protocol included tests that have since been removed. In the format presented above, the protocol would, therefore, be expected to last less than 4 h. For our study in this reduced format, the first 40 min consisted of finding the “*ideal*” electrode placement (see 2.1.1) and undertaking the “*tracking tasks*” (see 2.1.3). The “*reaction time tests*” for “*EMG skill*” analysis (see 2.1.2) took 20–30 min while the “*uncertainty tests*” (see 2.2.1 and 2.2.2) lasted a further 50–60 min. Finally, 40–50 min were spent setting up and undertaking the “*cylinder task*” (see 2.3). Breaks were provided at set points in the protocol to ensure participants’ attention was maintained.

### 3.2 Initial Analysis

#### 3.2.1 Reaction Time Tests for the Analysis of EMG Skill

**Figure 6:**
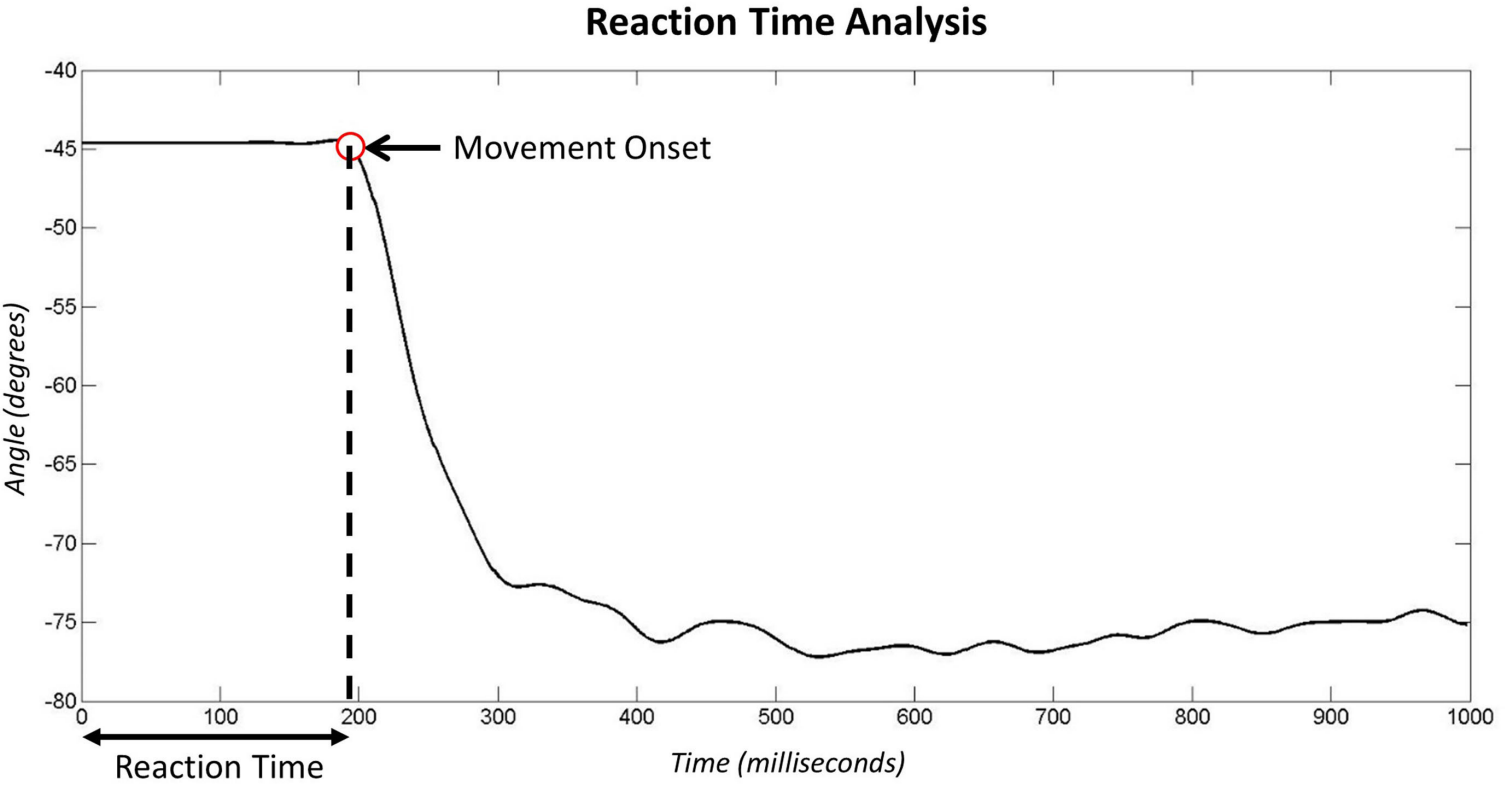
**Analysis of goniometer data recorded after the LED stimulus presentation in the reaction time experiment**. The red marker signifies the point identified as the onset of movement.

During the “*reaction time tests*” (see section 2.1.2), data were recorded from the goniometer both before and after the stimulus (LED) was presented. Figure 6 shows example data recorded during the second of stimulus presentation. The red circle identifies the time point identified as the moment of hand movement onset in response to stimulus presentation. More detail on the algorithms employed to identify movement onset are presented in the Supplementary Material.

**Figure 7:**
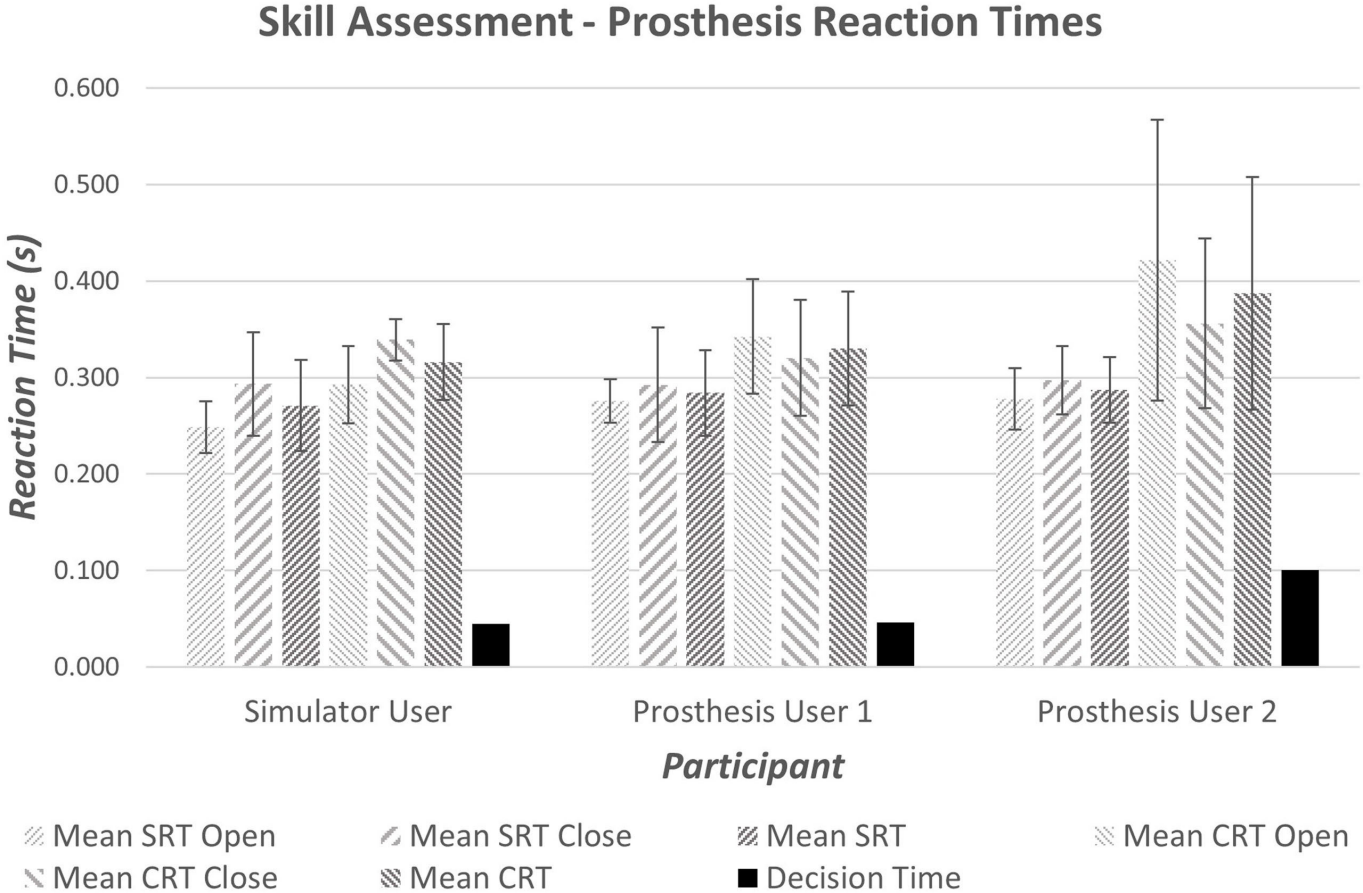
**Average simple and choice reaction times for the anatomically intact participant and prosthesis users**. The decision time is calculated as the difference between the mean CRT and the mean SRT.

It is widely accepted that the mean reaction time for college-aged individuals undertaking simple reaction time (SRT) tests with light-based stimuli is around 190 ms (0.19 s) (Kosinski and Cummings, 2004). During tests where the stimulus determines the reaction (CRT tests), times are often slower; exact speeds depend on the test. Moreover, the inherent delay introduced within the prosthesis would be expected to produce prosthesis reaction times that are longer than the anatomical reaction times. Initial results demonstrated measured SRT of 270–290 ms (Figure 7); furthermore, an increase in reaction time was seen between the Simple and Choice Reaction Times of 45–100 ms (“*Decision Time*”). It is worth noting that reaction times and consistency improve after first introduction to a new task (Kosinski and Cummings, 2004); this may show as a learning effect in the “*decision time*” over the small number of repeats. However, we decided not to randomize the order so that all participants underwent the same sequence of testing: the SRT first, then the CRT (see section 4). The “*decision times*” presented in Figure 7 suggest that Prosthesis User 2 was less skilled at deciding which muscles to activate than the other two participants.

#### 3.2.2 Tracking Tasks for the Analysis of EMG Skill

**Figure 8:**
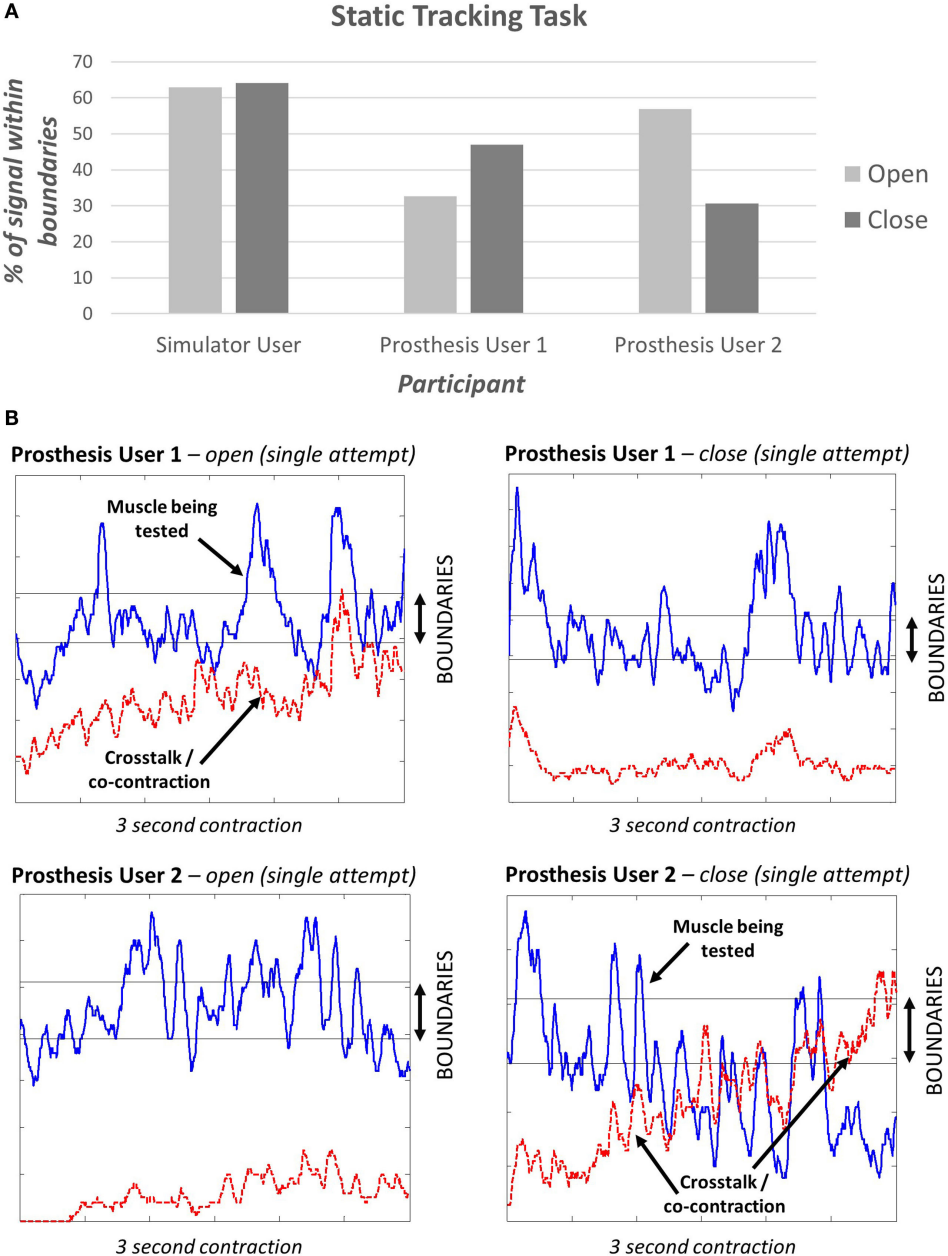
**(A) Results of the static tracking task**. Participants were provided with three opportunities to achieve their best signal. Here, we present the percentage of time the signal was within the boundaries over the 3-second period. **(B) Signals from the two prosthesis users** – the blue line is the signal being tested, the red dashed line shows the signal from the muscle that should remain relaxed.

The “*static tracking task*” (see section 2.1.3) assessed the participant’s ability to maintain a specified signal level. This task demonstrated that different levels of “*EMG skill*” can be measured and did not show a ceiling effect; i.e., no participant achieved 100% (Figure 8A). The simulator user appeared to perform better than either of the prosthesis users. It is interesting to note that during a sustained contraction, both prosthesis users demonstrated co-contraction or crosstalk for one of the two muscle groups (Figure 8B).

All participants were able to complete the “*dynamic tracking task*” (see section 2.1.3). Two participants performed better when only one car (muscle signal) was under assessment (Part 1), with a 20–40% higher success rate than when presented with 2 cars (Part 2). Prosthesis User 2, who demonstrated large amounts of co-contraction or crosstalk when activating the close signal (Figure 8B), did not fit this trend, instead a 10% improvement was seen in the success rate for the close signal for Part 2, and a 60% reduction in success with the open signal. During this second part of the dynamic task when two cars were being controlled, the participant was unable to relax the open signal while contracting the close muscle. This meant that the “open car” was guaranteed to “crash” for at least 50% of the gaps. It is possible that this participant, therefore, changed strategy to concentrate on the easier to control close signal. Alternatively, it is possible that this participant was unable to visually track the two cars and struggled with focusing equally on controlling each signal. One further suggestion is that this links with the reaction time results, which showed that this participant found deciding which muscle to activate harder than the other participants.

At this stage, it is not possible to draw any firm conclusions based on these results. However, we have demonstrated that both “*tracking tasks*” offer the possibility of differentiating between different levels of skill in controlling the EMG signal. Based on these tracking tasks, the simulator user demonstrated a higher level of skill than the two prosthesis users.

#### 3.2.3 Effects of Electrode Interface Condition on EMG Transduction

Both prosthesis users experienced some difficulty in completing the tasks designed to measure the extent of “*unpredictability*” in transduction of the EMG signal leading to “*uncertainty*” (“*uncertainty tests*,” see section 2.2). User 1 had a good level of control over the prosthesis, and was able to operate it as desired, however, the residual limb was very short. Consequently the participant found the addition of the 500 g mass fairly difficult to hold, reporting discomfort at the elbow. User 2 had a longer residual limb and reported feeling the additional load in his shoulder muscles. Both participants were happy to undertake the task with a 500 g load attached to the hand but would have struggled to support the prosthesis if the mass was much heavier.

**Figure 9:**
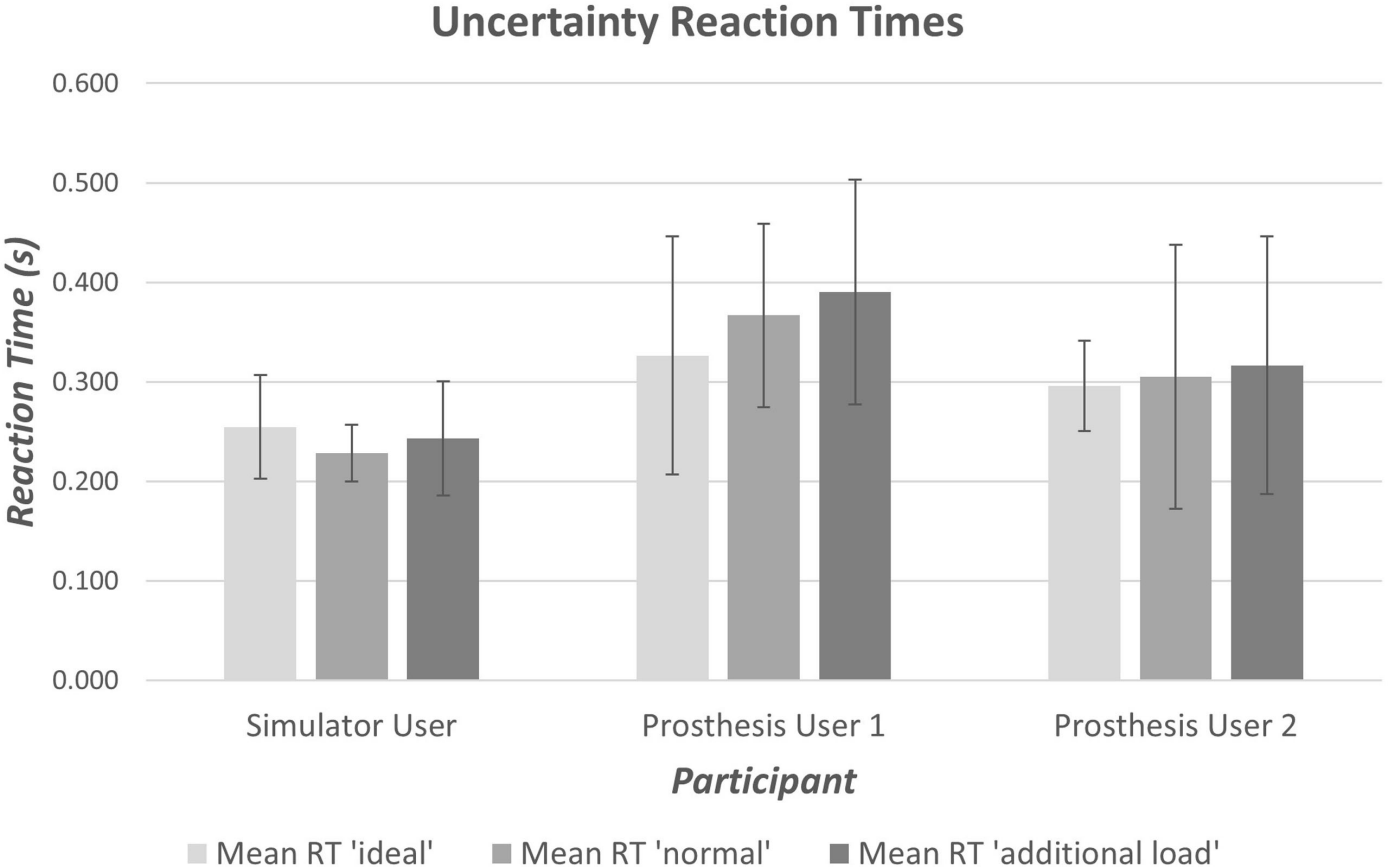
**Result of reaction time tests to assess “*unpredictability*” introduced at the electrode–skin interface, by the electrode fit**. Prosthesis User 2 demonstrates a larger amount of variability with the prosthetic socket than when using the “ideal” electrode contact setup with the electrodes bandaged to the limb.

**Figure 10:**
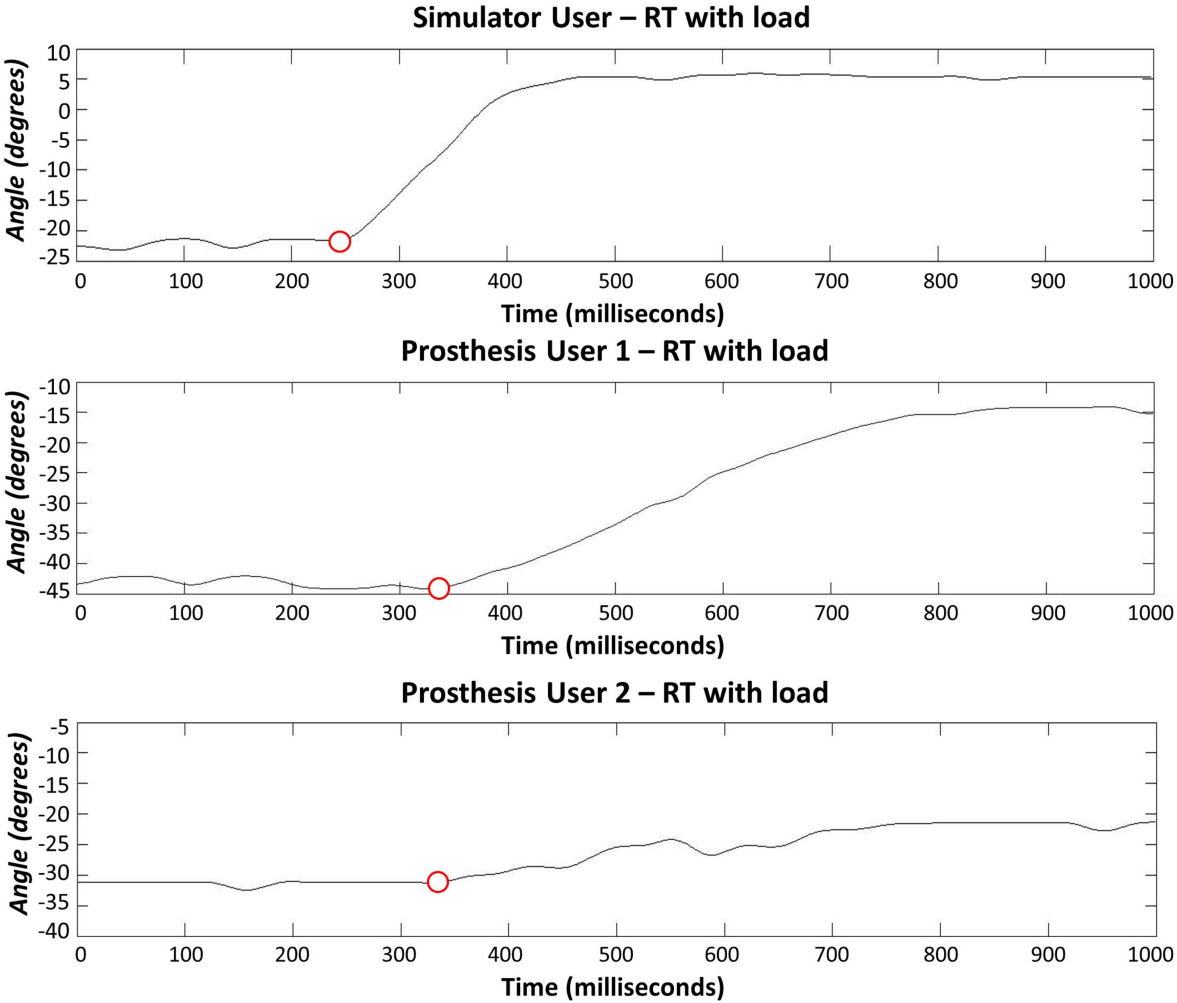
**Reaction times (hand opening) using the socket-housed electrodes with additional load added to the hand**. Prosthesis User 1 noticed slower movement of the hand with the addition of the load, whereas Prosthesis User 2 experienced a large amount of difficulty in overcoming the close function while trying to open the hand.

The anatomically intact participant using the simulator did not exhibit any clear difficulty with completing the reaction time portion of the task (Section 2.2.1), however, when moving between the arm postures (see section 2.2.2), four unwanted activations occurred (two with the “*ideal*” interface condition and two with the “*additional load*”). The reaction time data from Prosthesis User 1 showed a large amount of variation in reaction times for all three interface conditions (Figure 9); however, this user did not experience any unwanted activations of the hand. Finally, Prosthesis User 2 only experienced a small amount of variability in reaction times (Figure 9) when undertaking the task with the “*ideal*” electrode interface condition (electrodes bandaged to the limb). However, when the socket was introduced (“*normal*” interface condition and “*additional load*”), the participant encountered a large amount of difficulty in getting the prosthesis to react as desired. For 13 of the 20 open tasks, the hand closed when the participant attempted to open it; and for those tasks where the participant did manage to open the hand, the movement trajectory was not smooth. Figure 10 shows a comparison of the goniometer data between Prosthesis User 2 and the other two participants. It is worth noting that each participant used a different prosthetic hand for this assessment and that the total aperture for the hand used by Prosthesis User 2 was much smaller than for the other two participants, hence the difference in range. Moreover, Prosthesis User 2 had a much looser socket fit than User 1. Consequently, as the “open muscle” contracted, the limb seemed to push against the socket moving the “close electrode” away from the skin and activating the close movement instead. This “*unpredictability*” in socket fit was also highlighted by the seven unwanted activations when transitioning between the different arm positions.

#### 3.2.4 Functionality Assessment

All participants began with the medium difficulty task (“*task B*”); completion of the task ranged from 100% (Prosthesis User 1) to less than 50% (Prosthesis User 2) of trials. Both Prosthesis User 1 and the simulator user completed over 80% of trials of “*task B*” and, therefore, moved on to the harder task (“*task C*”). Prosthesis User 2 experienced difficulty grasping the cylinder, and often dropped it as he rotated it to the horizontal. When attempting the easier task (“*task A*”), he completed 90% of the trials; however, during two of these trials, he missed the cylinder on the first attempt of “reach-to-grasp.”

**Figure 11:**
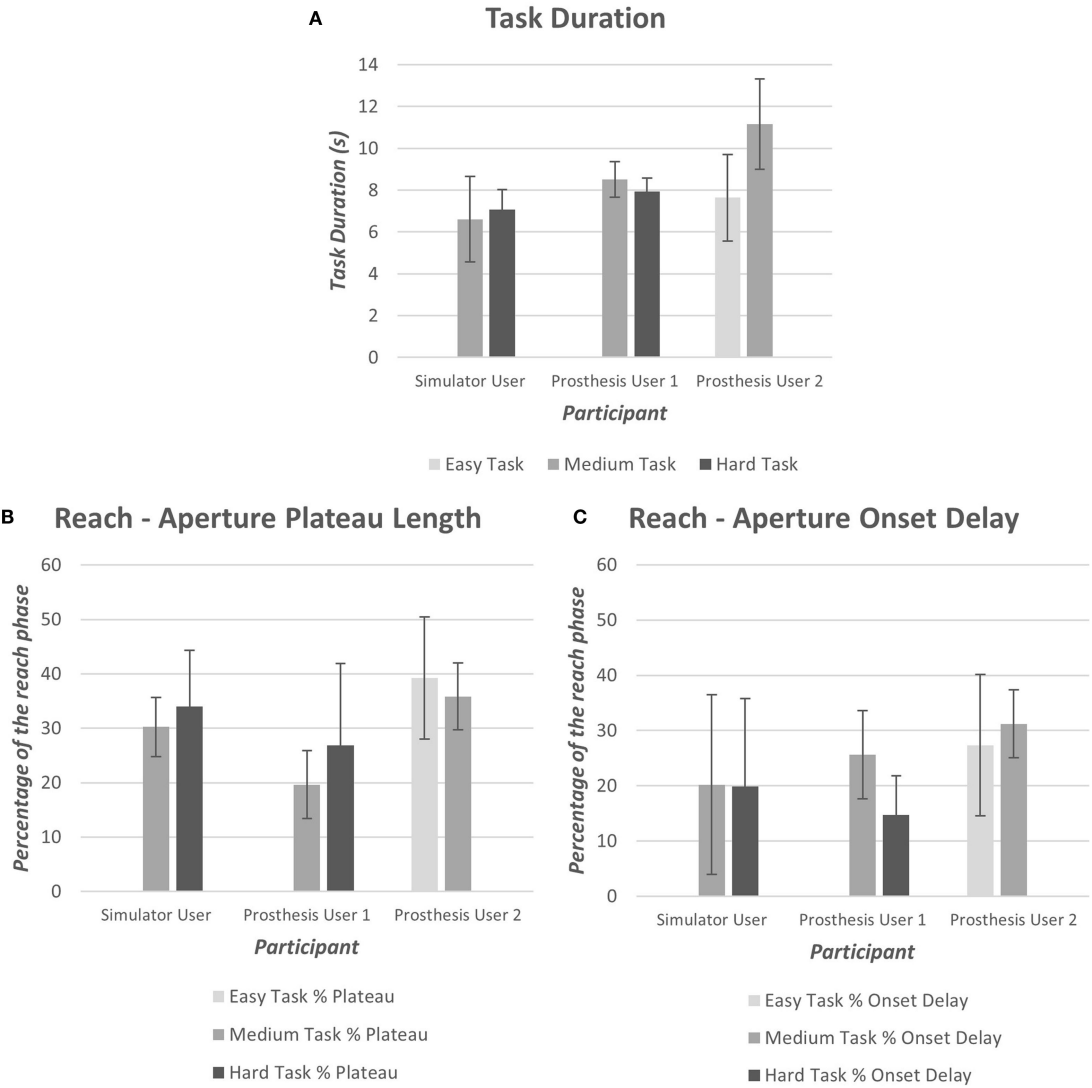
**(A) Mean task duration for each of the difficulty levels (Easy “A,” Medium “B,” and Hard “C”), (B) mean aperture “reach plateau” length as a percentage of the reach phase, (C) mean aperture onset delay as a percentage of the reach phase**.

As introduced in Section “2.3.1,” data were collected using wrist and chest-mounted IMUs, an electronic goniometer and an eye tracker. The systems were synchronized using the button press; pilot data demonstrated that synchronization was successful. The task durations, based on the button timestamps, illustrate that Prosthesis User 2 performed the medium difficulty task (“*task B*”) at a slower rate than the other two participants (Figure 11). Prosthesis User 1 was the most consistent regarding the time taken to perform the task, and as noted above, the most successful. Furthermore, Prosthesis User 1 demonstrated aperture patterns more similar to the healthy norms with a shorter “*reach plateau*” in the reach phase (Figure 11); onset delay was similar across the three participants (Figure 11).

**Figure 12:**
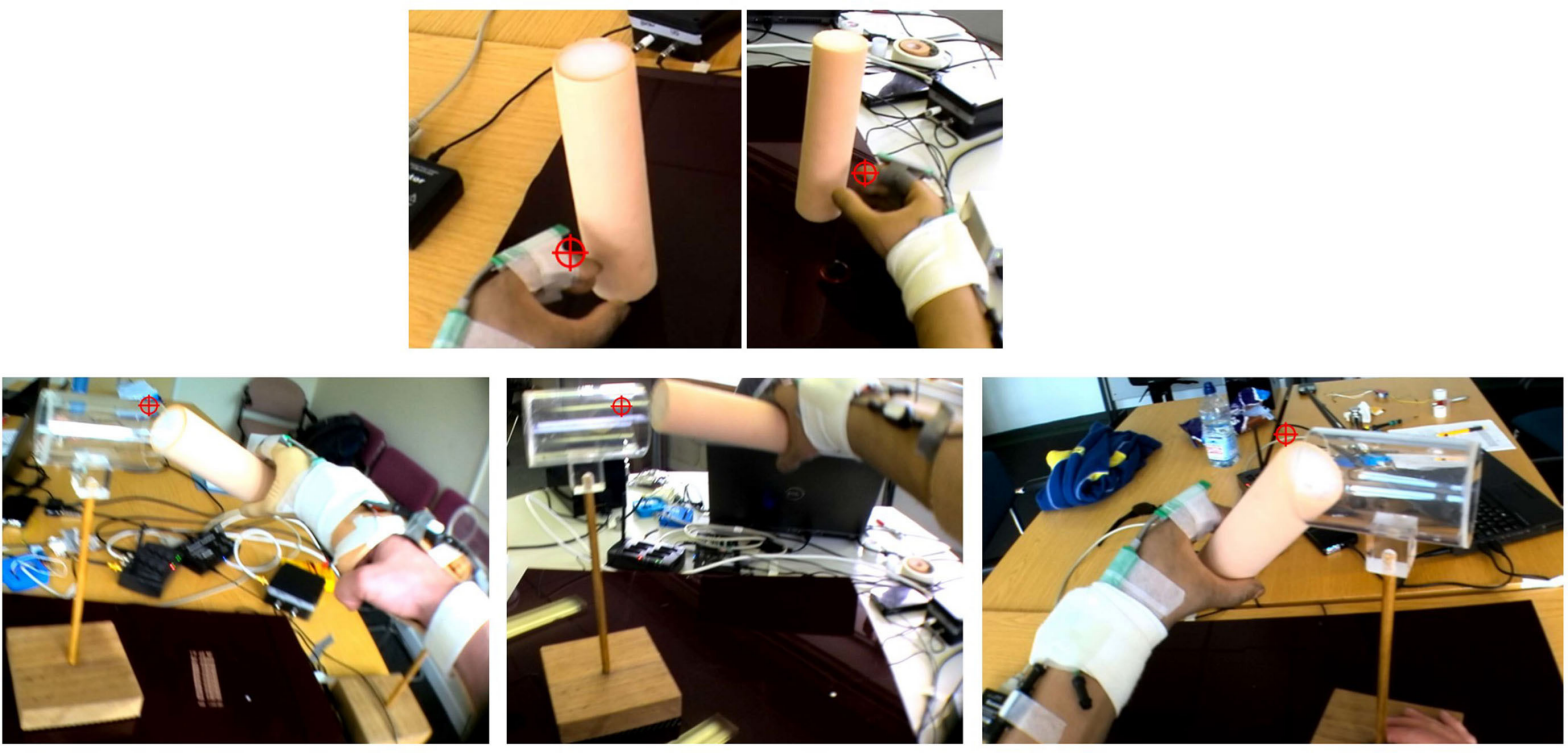
**Example eye tracking video – the crosshair shows the point of gaze fixation**. Top: both Prosthesis Users looked at the hand at a point in the reach to check their hand aperture. Bottom: the different strategies employed to complete “task B” can be seen – left: simulator user, middle: Prosthesis User 1, and right: Prosthesis User 2 – Prosthesis User 2 struggled to complete this task and would drop the cylinder when the arm was brought to the horizontal, therefore, he delayed this movement.

**Figure 13:**
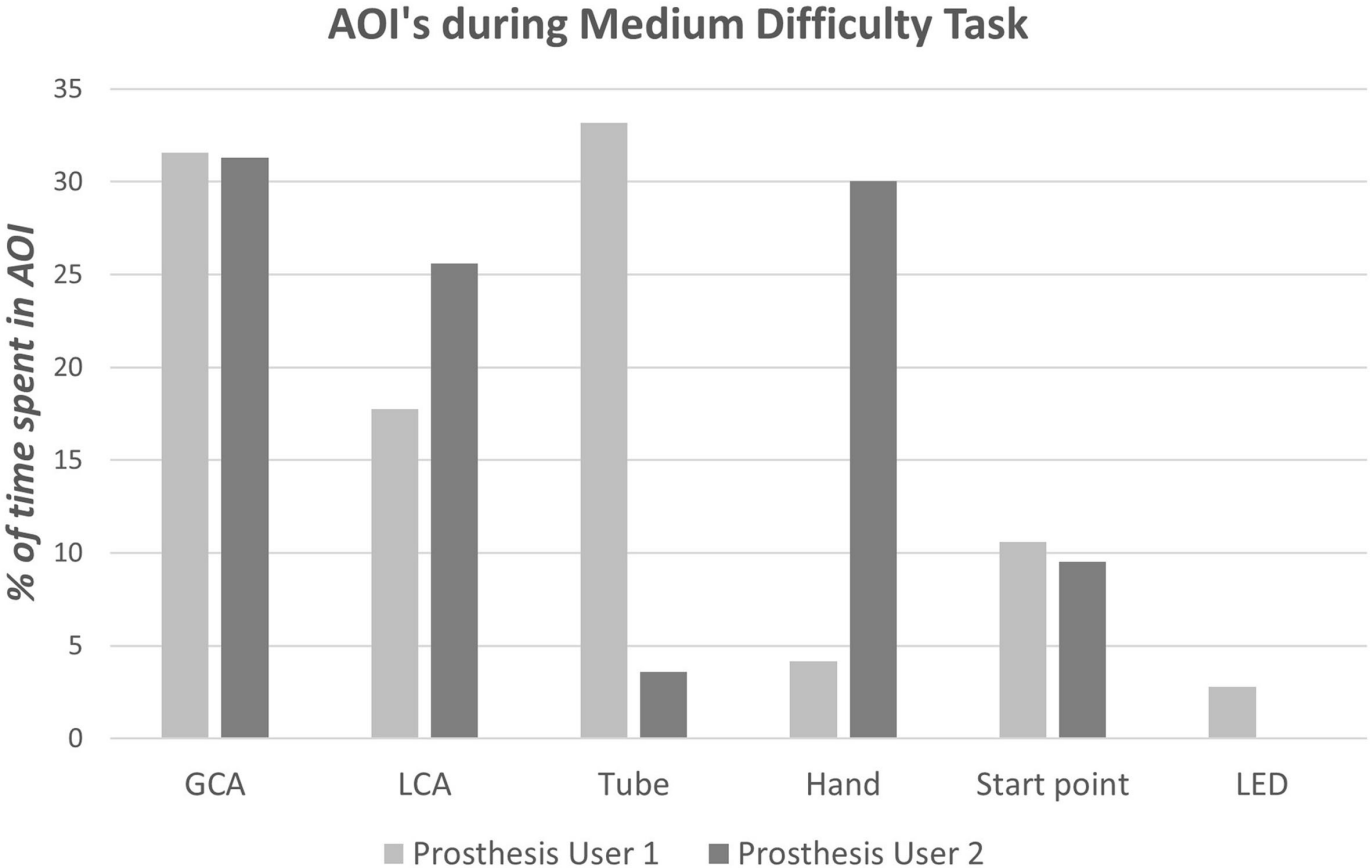
**Results of the gaze analysis for the first successful trial of the medium difficulty task (“task B”) for each of the prosthesis users**.

As highlighted above, Prosthesis User 2 struggled to complete “*task B*,” dropping the cylinder during rotation of the arm; the screenshots in Figure 12 summarize the technique employed by the participant to overcome this “*unpredictability*.” Unlike Prosthesis User 1 and the simulator user, Prosthesis User 2 waited until the last minute, when the cylinder was in contact with the tube, before rotating the cylinder to the horizontal. The participant’s “*uncertainty*” as to how the hand would respond is highlighted in the results of the eye tracking. The eye tracking videos (Figure 12) were individually coded frame by frame to establish where the participant was looking. As can be seen in the images at the top of Figure 12, both prosthesis users looked at their hand during “reach-to-grasp,” however as can be seen in Figure 13, there were noticeable differences in the gaze patterns of these two users. Prosthesis User 2 spent the majority of the time looking at the hand and the cylinder, tracking its movement, while Prosthesis User 1 showed a higher level of confidence in the hand, looking ahead to the cylinder and the tube. During the “reach-to-grasp” component of the task, Prosthesis User 1 looked ahead of the hand for 76% of the time, while Prosthesis User 2 relied on looking at the hand for over 50% of the time.

#### 3.2.5 Everyday Usage

**Figure 14:**
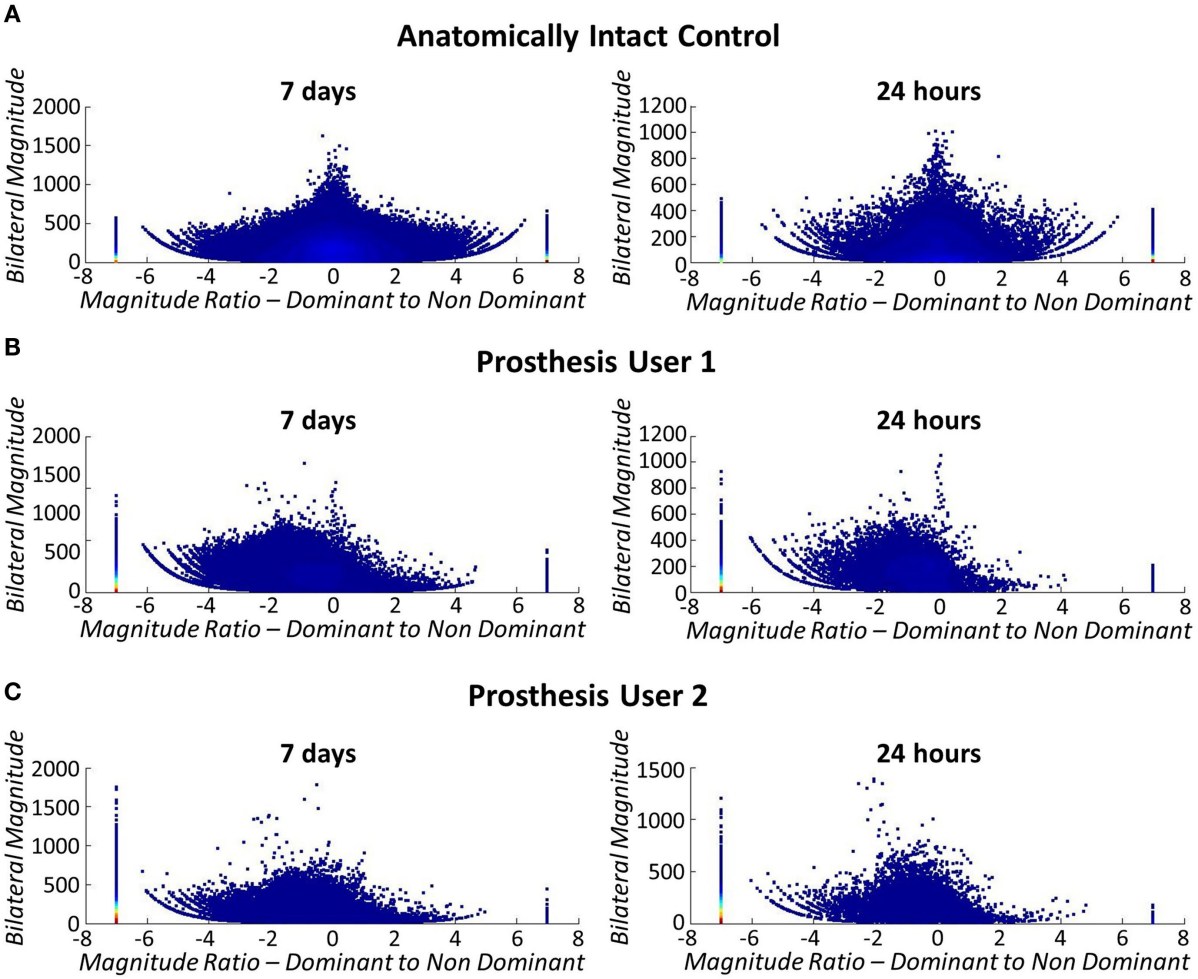
**Bilateral arm use (left: 7 days, right: 24 h)**. The stack at −7 signifies unilateral dominant arm use (anatomical arm), +7 signifies unilateral non-dominant arm use (prosthesis), and 0 signifies both limbs contributing to activity at the same level. Each marker represents 1 s of data and the color density is a count of the number of data points. **(A)** Top: bilateral arm use for anatomically intact control subject. Arm use is symmetrical across both arms, regardless of limb dominance. **(B)** Middle: bilateral arm use for Prosthesis User 1. **(C)** Bottom: bilateral arm use for Prosthesis User 2.

As explained in Section “2.4” participants were asked to undertake activity monitoring over the period of 1 week. For the purposes of this pilot study, data were only collected for the two prosthesis users; however, to check the methods against Bailey’s data (Bailey et al., 2015) (see section 2.4), one separate anatomically intact participant underwent activity monitoring using their anatomical arms. The anatomical results echoed Bailey’s findings with symmetrical use across the two arms represented by a median “*magnitude ratio*” of 0.11 (IQR = 3.28) (Figure 14A).

At present, no algorithm exists allowing for differentiation between non-wear and passive-use of the prosthesis using the wrist worn Actigraph monitors. Consequently, participants were asked to complete activity diaries, which subsequently showed that Prosthesis User 2 only wore his device for 3 of the 7 days, while User 1 wore his all week. This non-wear is reflected in the activity monitor data with purely unilateral use of the anatomical arm on these days and no activity counts for the prosthesis. From the activity diaries, we know that both participants generally wore their prosthesis for 10 h or more on the days when they were worn. It is, therefore, important that the data are collected over the week long period to ensure that representative data for each user is collected.

Figures 14B,C illustrate that both prosthesis users rely on their anatomically arm to a greater extent than the stroke patients participating in Bailey’s study (Bailey et al., 2015). Both prosthesis users demonstrated median “*magnitude ratios*” of −7 [IQR = 5.40 (Participant 1) IQR = 0 (Participant 2)] (unilateral use of the intact arm) similar to the group of Bailey’s stroke participants who rely most on their non-paretic arm. However, when only the data collected while the prosthesis was worn is included in the comparison, the median “*magnitude ratios*” reduce to −2.55 (IQR = 6.42) for Prosthesis User 1, and −2.42 (IQR = 6.76) for Prosthesis User 2. It is interesting to note that although Prosthesis User 1 wore the device for more hours during the week, both participants demonstrated similar median “*magnitude ratios*.” Furthermore, it is notable that the “*bilateral magnitude*” of User 1’s activity was of a level much closer to the stroke patients, while User 2 demonstrated activity to the same magnitude as Bailey’s healthy controls.

## 4 Limitations and Future Work

For the purposes of this study, the prosthesis control chain has been characterized up to the point of EMG signal transduction. In reality, however, further to the “*EMG skill*” and “*uncertainty*” addressed above, an inherent electromechanical delay will be introduced by the prosthesis itself, which will also impact on the ease of controlling the device. This delay is a culmination of the delays introduced through processing of the EMG signals and stiction/backlash in the prosthetic hand mechanisms. The measurement of these delays is complex requiring artificial activation of the electrodes. Previous work has been undertaken to calculate the optimal controller delay in a prosthesis (Farrell and Weir, 2007); however, the delays in clinically available prostheses are not available. Future work would involve integrating the measurement of electromechanical delays into the protocol detailed above.

Other limitations are the assumptions that have been made with respect to the “*Reaction Time Tests*” (Section 2.1.2). Reaction time experiments involving simple and choice reaction times would normally be randomized, and undertaken in large numbers, to overcome learning or attentional effects. This study involves the comparison of performance in these tasks between participants, therefore, it is important that all participants experience the same tasks in the same order. Furthermore, time constraints limit the number of repeats that can be undertaken. Although different participants may learn at different rates, it is assumed that as the task is novel to all participants the results will be comparable.

Furthermore, the tube used in the “*cylinder task*” is transparent, meaning that when the cylinder is within the tube it can be difficult to identify whether the participant is looking at the cylinder or the tube (likely both). Similarly when the gaze is on the GCA of the cylinder, as the hand approaches and blocks the view, it is not clear whether the AOI should be coded as the hand or the GCA. An inter-rater reliability study will be undertaken to assess the proposed approach to coding the gaze data.

As discussed in Section “2.4,” the analysis methods for the assessment of everyday upper limb use were borrowed from the study by Bailey et al. (2015). A limitation of this method is that it does not inform on actual hand use. Therefore, it is not possible to confirm whether the activity counts recorded relate to the prosthetic hand being used in an active or passive manner. For future studies, it would, therefore, be worth including a system to also monitor hand movements. This approach was advocated by Sobuh et al. (2010) and a recent paper by Rowe et al. (2014) demonstrated the potential for a similar approach in the monitoring of anatomically intact upper limb movements.

Finally, reliability and validity of the experimental setups and corresponding outcome measures need yet to be explored. Reliability can be established through a test–retest study in a subset of our planned cohort. Validity of measures, where possible, may be investigated via comparison to related, established measures, for example, by comparing functional measures during “*cylinder task*” performance to SHAP and/or Box and Blocks test scores. For validation of measures characteristic of prosthesis control, we may utilize a known-groups assessment to investigate their sensitivity to distinguish between novice and experienced myoelectric prosthesis users, and we could further conduct a responsiveness study in novice myoelectric prosthesis users to identify whether an individual measure of prosthesis control responds to effects of training to perform the corresponding experimental set up of the protocol.

## 5 Conclusion

In this paper, we presented a protocol for the assessment of user skill in controlling EMG signals (“*EMG skill*”) and “*unpredictability*” in the acquisition of these signals. These are to be assessed against overall user “*functionality*” and “*everyday usage*” of the myoelectric prosthesis. To demonstrate the protocol, results of initial pilot work were presented.

Pilot work and initial analysis of the results suggest that this protocol will be able to successfully identify differences in the “*EMG skill*” level of participants and characterize the “*unpredictability*” at the electrode interface. Data have been successfully collected for each aspect of the functional task that will allow analysis of how each control factor affects “*functionality*.” Furthermore, analysis of the activity monitoring data will allow assessment of control factors against “*everyday usage*.”

Although the results presented are not sufficient to draw firm conclusions, Prosthesis User 2 appeared to demonstrate a lower level of “*functionality*” than User 1, which could be attributed to either of the control factors at this stage. By collecting data across a larger cohort of prosthesis users, it should be possible to identify the relative contributions of these factors.

Finally, although the protocol is relatively long, pilot participants were provided with regular breaks and were happy with the distribution of the tasks; the length of the study was not felt to be excessive. Performing all tasks in a single test session (including breaks to avoid fatigue) has the advantage that it facilitates protocol completion in myoelectric prosthesis users, who are largely part of the working population and, hence, could prove difficult to schedule on multiple occasions within a reasonable time frame. Nevertheless, each experimental setup has been designed in such a way that it could be performed in isolation of other parts of the protocol, providing useful insights on the isolated factor the experiment is concerned with. Hence, while the complete protocol may be predominantly used by researchers due to its complexity, individual parts could be adopted by clinicians to support their decision making.

## Ethical Considerations

Ethical approval for the work reported here was granted by the University of Salford ethics committee (*REF: HSCR 15-130*). All participants provided informed written consent.

## Dissemination of Results

It is intended that the results of the main study will be published in peer-reviewed journals and will be uploaded as part of the lead author’s PhD thesis onto the University of Salford SEEK website. Additionally, it is intended that the results of this early work and the final study will be presented at conferences both in the UK and internationally.

## Author Contributions

The study was initially conceived by LK. All authors contributed to the design of the study, which was implemented by AC. All authors were involved in the drafting and approving of the manuscript.

## Conflict of Interest Statement

The authors declare that the research was conducted in the absence of any commercial or financial relationships that could be construed as a potential conflict of interest.

## Footnotes

^1^The manufacturers do not disclose the details of the scale used to represent signal amplitude, hence, the units are not reported.

^2^The trunk sensor is used for setting up the cylinder tasks; the distance of the cylinder from the resting hand position should allow the participant to reach the cylinder without leaning forwards. The trunk sensor will also record trunk compensatory movements during task performance.
