# Corrigendum: The Reality of Myoelectric Prostheses: Understanding What Makes These Devices Difficult for Some Users to Control

**DOI:** 10.3389/fnbot.2018.00015

**Published:** 2018-04-03

**Authors:** Alix Chadwell, Laurence Kenney, Sibylle Thies, Adam Galpin, John Head

**Affiliations:** Centre for Health Sciences Research, University of Salford, Salford, United Kingdom

**Keywords:** prosthesis, myoelectric, activity monitoring, control, upper limb, functionality assessment

In the original article, there was an error. The equation for the Magnitude Ratio presented in the Methods and Analysis was incorrect and should be written *ln*(*VM*_*NonDom*_/*VM*_*Dom*_).

A correction has been made to Methods and Analysis, Everyday usage (Section 2.4), paragraph 1:

Current methods of quantifying everyday prosthesis use involve self-report (Roeschlein and Domholdt, 1989; Sherman, 1999; Gallagher and MacLachlan, 2000; Raichle et al., 2008), which is known to be prone to recall and bias errors (Metcalf et al., 2007; Brown and Werner, 2008). Accelerometer-based activity monitoring (Noorkõiv et al., 2014) provides an opportunity to observe actual prosthesis use outside of the clinical environment; however, to date no studies have been published on a cohort of upper limb prosthesis users. We have adapted a protocol developed for stroke patients (Bailey et al., 2015). This research involved participants wearing an activity monitor (Actigraph GT3X+) on each of their wrists while they went about their normal daily activities. The Actigraph monitors provide continuous logging of raw accelerometer data (sampled at 30 Hz). The data are downloaded using proprietary software, filtered, and down sampled to 1 Hz. The processed data are expressed as activity counts (0.001664 g/count) (Actigraph Corp., 2015), which are converted into vector magnitudes (sum of the counts along each axis $\sqrt{x^{2}+y^{2}+z^{2}}$). For each second of data, Bailey et al. (2015) combined the vector magnitudes from each of the two wrist worn monitors (dominant and non-dominant arm) to inform on the magnitude of activity across both arms, expressed as the “*bilateral magnitude*” (*VM*_*Dom*_ + *VM*_*NonDom*_), and the contribution of each arm to the activity, expressed as the “*magnitude ratio*” [*ln*(*VM*_*NonDom*_/*VM*_*Dom*_)].

The authors apologize for this error and state that this does not change the scientific conclusions of the article in any way.

## Conflict of interest statement

The authors declare that the research was conducted in the absence of any commercial or financial relationships that could be construed as a potential conflict of interest.
