# Supplementary material for: The Reality of Myoelectric Prostheses: Understanding What Makes These Devices Difficult for Some Users to Control
Source: Front Neurorobot. 2016 Aug 22;10:7. doi: 10.3389/fnbot.2016.00007 (PMC4992705; doi:10.3389/fnbot.2016.00007)
Supplement: Supplementary file 1 [file Data_Sheet_1.DOCX]

Supplementary Material

Identification of movement onset based on goniometer data

Alix Chadwell^1^, Laurence Kenney^1*^, Sibylle Thies ^1^, Adam Galpin ^1^, John Head ^1^

^1^Centre for Health Sciences Research, University of Salford, UK

*** Correspondence:**Laurence Kenney
l.p.j.kenney@salford.ac.uk

# Methods for the identification of movement onset and exclusion of incorrect responses

Algorithms, described below, have been developed which examine the data recorded from the goniometer to identify, early reactions, incorrect reactions and to calculate the reaction time.
