# Supplementary material for: The Reality of Myoelectric Prostheses: Understanding What Makes These Devices Difficult for Some Users to Control
Source: Front Neurorobot. 2016 Aug 22;10:7. doi: 10.3389/fnbot.2016.00007 (PMC4992705; doi:10.3389/fnbot.2016.00007)
Supplement: Supplementary file 2 [file Data_Sheet_2.DOCX]

Supplementary Material

Removal of non-wear time based on accelerometer data

Alix Chadwell^1^, Laurence Kenney^1*^, Sibylle Thies ^1^, Adam Galpin ^1^, John Head ^1^

^1^Centre for Health Sciences Research, University of Salford, UK

*** Correspondence:**Laurence Kenney
l.p.j.kenney@salford.ac.uk

# Methods for identification of prosthesis non-wear periods

As far as the authors can tell, there are no published detailed algorithms to distinguish wear time from non-wear time for wrist worn Actigraph GT3X+ monitor (Actigraph Corp) data. To address this, we first applied the approach taken by Bailey ([Bailey et al., 2015](#_ENREF_1)) to remove periods when the “*bilateral magnitude*” was equal to 0. This allowed exclusion of periods when it can be assumed that both activity monitors were removed. It appears that Bailey assumed that participants were either wearing both monitors, or none and hence no further analysis of wear were employed in Bailey’s study.

The monitoring of amputees introduces additional challenges to the ones faced by Bailey. The monitor worn on the prosthesis may be isolated from the anatomical upper limb by either removing the monitor from the wrist of the prosthesis, or removing the socket (with the monitor still attached) from the anatomical residual limb, so it is difficult from the monitor data alone to distinguish prosthesis non-wear time from monitor non-wear time. However, participants were asked to keep the monitor on the prosthetic socket throughout and, unlike the anatomical limb, there would be no obvious reason why the participants would not comply with this; it is reasonable to assume that, when bathing, showering, or sleeping, the prosthetic socket – not the monitor - would be removed. The only exception to this was the last day of recording, where in one case the participant (Prosthesis User 2) removed the monitor in the morning to return it to us (Supplementary Figure 1). We also invited participants to complete an activity diary, recording sleep times, the times when the prosthesis was worn, and the times when the monitors were removed from either arm and this record was also used in the analysis, as described below.

To address the challenge of detecting periods when the prosthesis was not worn, we used both diary record and visual inspection of both the activity count data and “raw” accelerometer data. We used diary record to exclude periods where there was activity evident from the accelerometer data, but the diary indicated the prosthesis was not worn (e.g. final day’s data for Prosthesis User 2). We used visual inspection of the monitor data to identify periods where we believed the participant to have removed their prosthesis. As discussed above, we assumed that all participants would remove their prosthesis (if worn) prior to going to sleep. The supplementary figures below show the data recorded from both activity monitors. The red, green and blue lines show the raw accelerometer data measured in g (1g = 1 unit of gravity or 9.81m/s^2), whilst the black line represents the activity counts generated by the proprietary algorithm in the Actilife software (these have been divided by 100 to allow them to be plotted alongside the raw data). All data from the activity monitor worn on the anatomical hand has been shifted vertically upwards by 10g, to illustrate the synchronous data from the two activity monitors, one plotted above the other. Overnight removal of the prosthesis was identified as being the period from the last activity count registered on one day until the first count on the next day. Single, isolated activity count spikes during this period were ignored, such as the spike at 65 hours in Supplementary Figure 1.

Supplementary Figure 2 shows day 3 data in more detail. Although the activity diary did not provide information on removal of the prosthesis during the day, the raw data would suggest that the device was not always worn. The yellow bars in Supplementary Figure 2 represent the length of the ‘quiet’ periods for the prosthesis in minutes. We decided to label one of these periods as non-wear based on the raw data as there was no movement of the prosthesis for a period of 133 minutes (>2 hours), whilst the anatomical hand was still very active. As both arms exhibited similar accelerometer profiles, we did not label the131 minute long period of low amplitude activity at 44-46 hours as non-wear, even though the number of activity counts during this period was very low. It is possible that the participant was travelling during this period and therefore was very inactive.

None of the other periods marked in Supplementary Figure 2 were labelled as non-wear. For comparison, the raw accelerometer data from Prosthesis User 1 who wore the device every day is presented in Supplementary Figure 3.

# References

Bailey, R.R., Klaesner, J.W., and Lang, C.E. (2015). Quantifying real-world upper-limb activity in nondisabled adults and adults with chronic stroke. *Neurorehabilitation and Neural Repair* 29**,** 969-978.


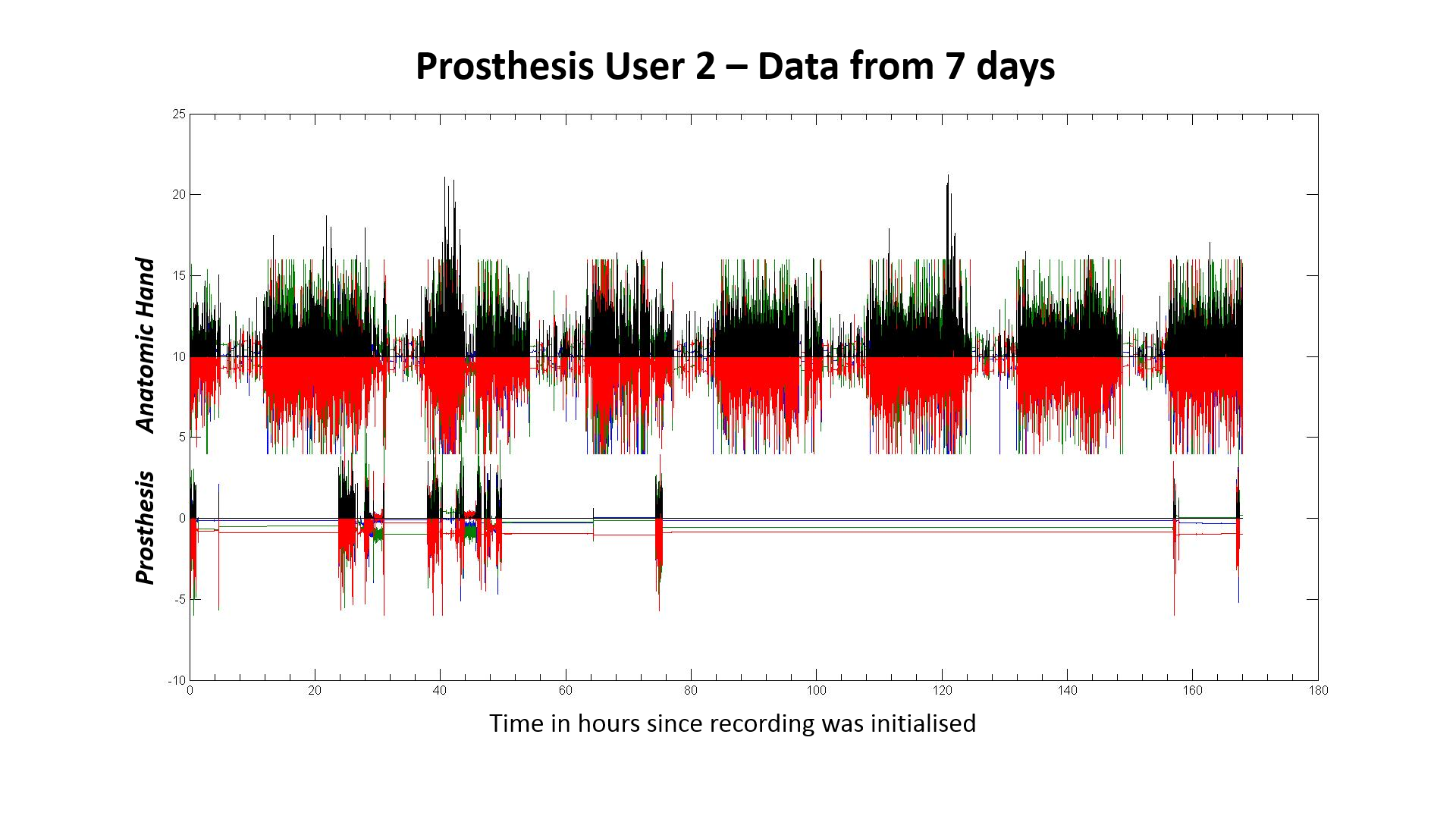


Supplementary Figure 1. Activity Monitoring Data from 1 week of prosthesis wear for Prosthesis User 2. The red, blue and green lines show the raw accelerations in g, whilst the black lines signify the activity counts (divided by 100 to scale). The data for the anatomic hand has been shifted upwards by 10g for visual purposes.


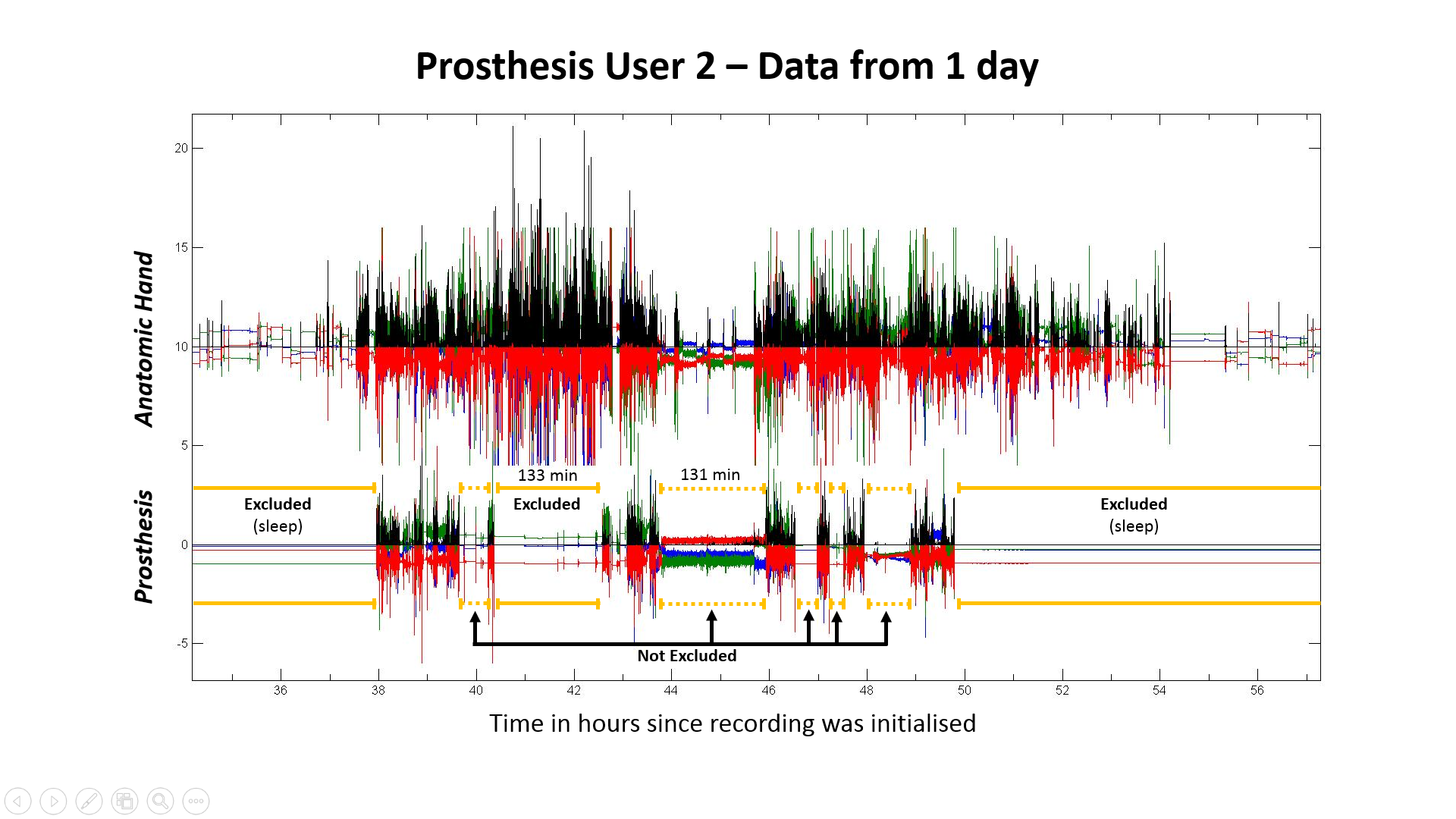


**Supplementary Figure 2.** Activity Monitoring Data from day 3 of prosthesis wear for Prosthesis User 2. The red, blue and green lines show the raw accelerations in g, whilst the black lines signify the activity counts (divided by 100 to scale). The data for the anatomic hand has been shifted upwards by 10g for visual purposes. The yellow bars show periods where the prosthesis was very inactive; solid lines represent the periods which were treated as non-wear time. 1 period during the day was excluded, all other periods were left in and assumed to be passive wear.


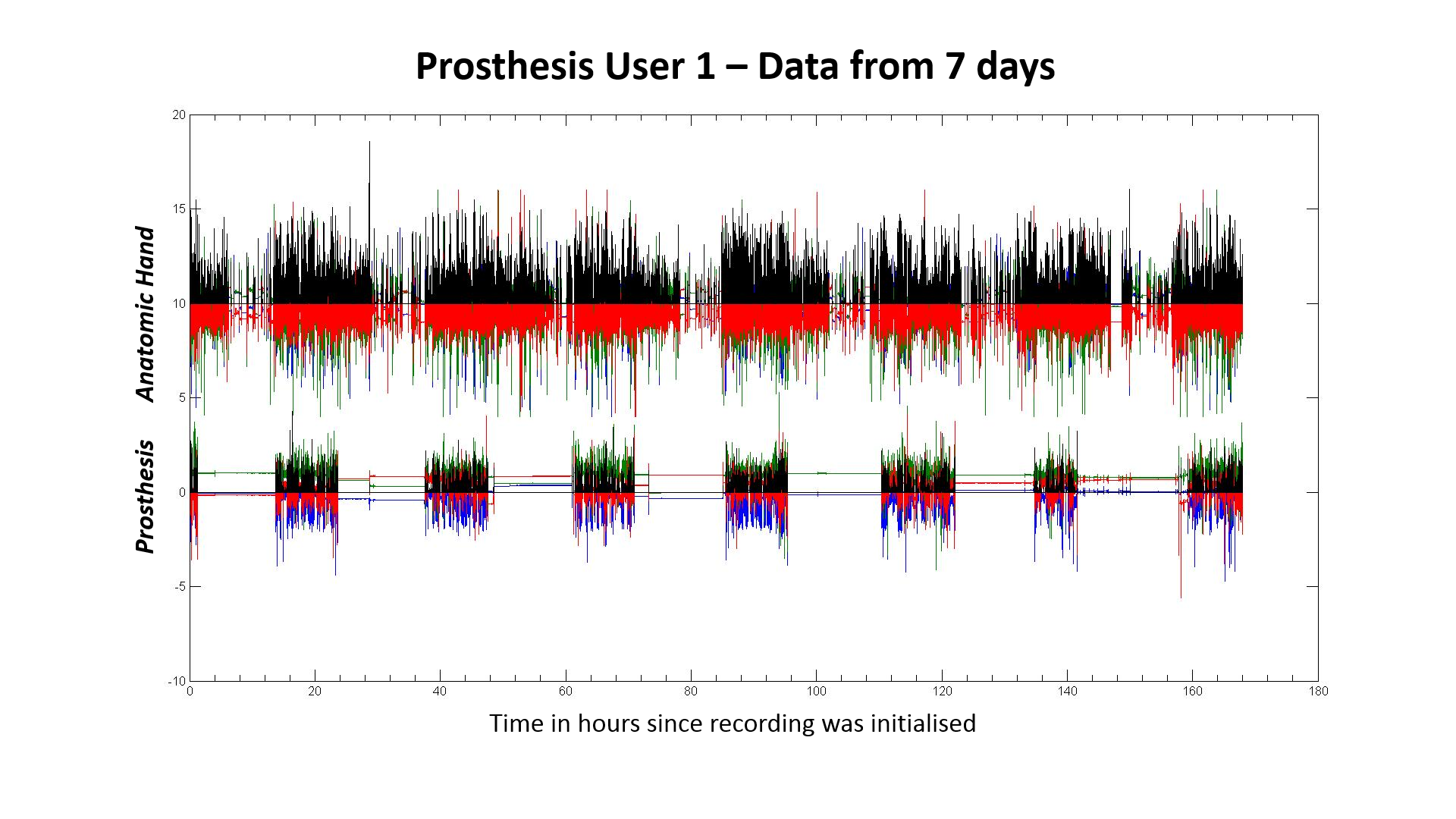


Supplementary Figure 3. Activity Monitoring raw acceleration data (measured in g) from 1 week of prosthesis wear for Prosthesis User 1. The data for the anatomic hand has been shifted upwards by 10g for visual purposes.
